# Rationalizing the Unprecedented Stereochemistry of an Enzymatic Nitrile Synthesis through a Combined Computational and Experimental Approach

**DOI:** 10.1002/anie.202017234

**Published:** 2021-07-21

**Authors:** Hilmi Yavuzer, Yasuhisa Asano, Harald Gröger

**Affiliations:** ^1^ Chair of Industrial Organic Chemistry and Biotechnology, Faculty of Chemistry Bielefeld University Universitätsstraße 25 33615 Bielefeld Germany; ^2^ Biotechnology Research Center Toyama Prefectural University 5180 Kurokawa Imizu Toyama 939-0398 Japan

**Keywords:** aldoxime dehydratase, chiral nitriles, enantioselectivity, enzyme models, rational protein design

## Abstract

In this contribution, the unique and unprecedented stereochemical phenomenon of an aldoxime dehydratase‐catalyzed enantioselective dehydration of racemic E‐ and Z‐aldoximes with selective formation of both enantiomeric forms of a chiral nitrile is rationalized by means of molecular modelling, comprising in silico mutations and docking studies. This theoretical investigation gave detailed insight into why with the same enzyme the use of racemic E‐ and Z‐aldoximes leads to opposite forms of the chiral nitrile. The calculated mutants with a larger or smaller cavity in the active site were then prepared and used in biotransformations, showing the theoretically predicted decrease and increase of the enantioselectivities in these nitrile syntheses. This validated model also enabled the rational design of mutants with a smaller cavity, which gave superior enantioselectivities compared to the known wild‐type enzyme, with excellent E‐values of up to E>200 when the mutant OxdRE‐Leu145Phe was utilized.

## Graphical abstract

Aldoxime dehydratases are able to enantioselectively dehydrate *E*‐ and *Z*‐aldoximes to give the opposite enantiomeric forms of a chiral nitrile. This stereochemical phenomenon has been rationalized by means of molecular modelling. When calculated enzyme mutants with modified active sites were prepared and used in biotransformations, they showed the theoretically predicted change in enantioselectivity in these nitrile syntheses.

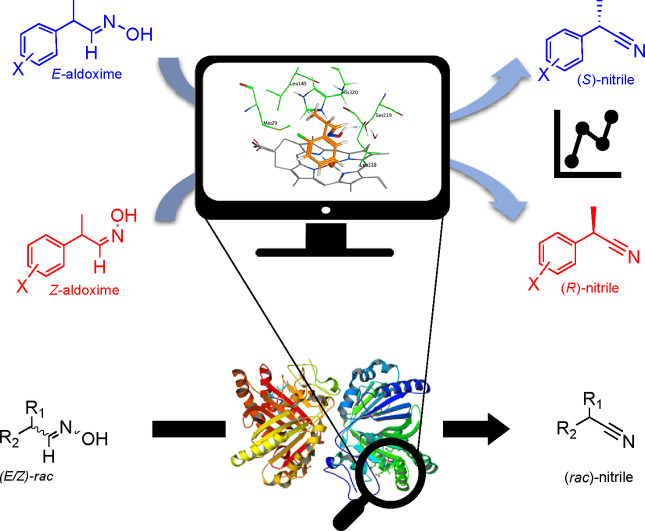

## Introduction

Designing stereochemical processes is of utmost importance for access to chiral building blocks needed for the production of chiral drugs.[^1^, ^2^, ^3^] While over the last decades numerous impressive examples with chiral chemocatalysts as well as enzymes, both representing fascinating chiral catalysts,[^1^, ^2^, ^3^] exist, at the same time “by definition” there is one general limitation for any type of stereochemical process. When starting from one (enantio)selective catalyst and a specific substrate (which can be either prochiral or racemic), only one enantiomeric form of the product will be obtained. This is true for any type of asymmetric catalysis starting from prochiral substrates as well as for resolutions starting from racemic substrates.[^1^, ^2^, ^3^] In other words, when having only one enantiomerically pure form of a chiral catalyst in hand, only one enantiomeric form of the product will be accessible in the corresponding transformation. This, however, can turn out as a limitation in particular for the field of biocatalysis,^[3]^ since only one enantiomeric form of the proteins (based on the l‐amino acids) is available, whereas the mirror image based on d‐amino acids is not formed in nature (although it should be added that in practice such a mirror image protein might not necessarily be required as often other proteins leading to the opposite enantiomeric form of a product exist).^[3]^

**Scheme 1:**
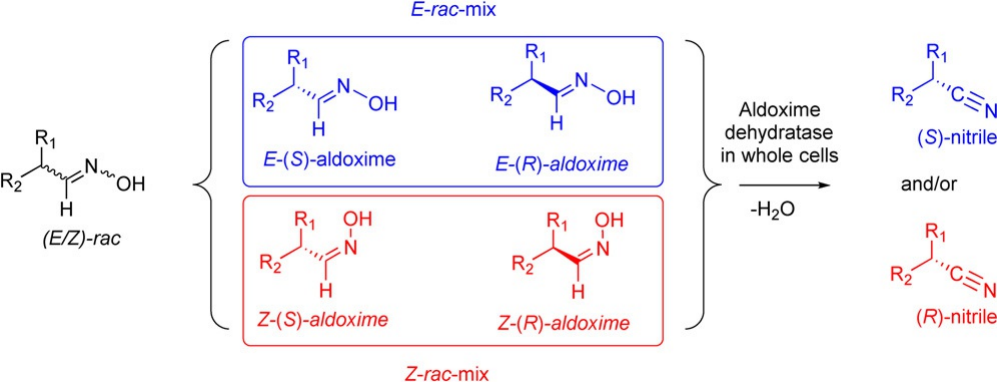
Enantioselective dehydration of aldoximes giving the corresponding nitrile.

However, very recently we identified a unique enzymatic transformation which gives access to both enantiomeric forms of a chiral nitrile although starting from the *same* racemic aldehyde and utilizing the *same* enzyme.^[4]^ This unprecedented stereochemical phenomenon turned out to have its origin in the formation of racemic *E*‐ and *Z*‐aldoximes as intermediates by simple condensation of the aldehyde as starting material and hydroxylamine. These racemic *E*‐ and *Z*‐aldoximes serve as the “real” substrate for the enzyme and are enantioselectively dehydrated to the chiral nitrile. It is noteworthy that the enantiopreference of this enzymatic resolution then surprisingly does not depend on the absolute configuration of the stereogenic center at the aldoxime, but on the *E*‐ or *Z*‐conformation of the aldoxime. In detail, in the presence of the same enzyme, using the *E*‐racemate as substrate then furnished the (*S*)‐nitrile, whereas the (*R*)‐nitrile was formed when starting from the *Z*‐racemate (Scheme 1).^[4]^ Thus, when separating these *E*‐ and *Z*‐aldoximes, both racemates undergo dehydration with formation of the opposite enantiomers of the nitrile products (whereas using the non‐separated racemic *E*/*Z*‐mixtures would lead to more or less racemic nitrile products).

The biocatalysts being capable of this unique transformation are aldoxime dehydratases.[^5^, ^6^] These types of enzymes were found about three decades ago, but still their number is limited and not much is known about them yet.[^7^, ^8^] A notable feature is their active site which combines the structural unit of a catalytic triad consisting of an arginine, histidine and serine with a heme moiety. From a practical point of view, it is noteworthy that the conversion of aldoximes to the corresponding nitriles by release of water proceeds without the need of any additional cofactor. In addition, the substrate scope proved to be very broad,[^4^, ^5^, ^6^, ^7^, ^8^, ^9^, ^10^, ^11^, ^12^, ^13^] and among the resulting aliphatic and chiral nitriles are various important products for the chemical industry.

To gain insight into this exciting and very unusual stereochemical behavior of this class of aldoxime dehydratases (which are at the same time attractive catalysts for chiral nitrile synthesis without the need for highly toxic cyanides), molecular modelling studies were performed, including in silico mutations and docking studies. In order to prove such data on rationalizing the selectivity of an aldoxime dehydratase, we combined these computational studies with laboratory experiments by preparing and characterizing the theoretically calculated enzyme mutants. Based on a general postulated mechanism for an aldoxime dehydratase by Nomura et al.^[14]^ we focused on rationalizing this unusual switch in enzyme selectivity by means of docking experiments. As a software, MOE (Molecular Operating Environment)^[15]^ was used, which is a powerful tool to find suitable ligand‐protein conformations. The software has already proven highly successful for a range of applications.[^16^, ^17^, ^18^, ^19^, ^20^, ^21^] With the combination of wet experiments including a variety of mutants and substrates we were able to understand this unique and unprecedented behavior regarding the enantioselectivity of these enzymes. Furthermore, based on this rational insight into the stereochemical properties of such enzymes, we were capable to calculate mutants with superior and dramatically increased enantioselectivities.

## Results and Discussion

In order to characterize the stereoselectivity of the aldoxime dehydratase from *Rhodococcus* sp. N‐771 (OxdRE‐WT)^[23]^ for model substrates as a basis for the molecular modelling studies, at first *ortho*‐, *meta*‐ and *para*‐fluoro‐substituted phenylpropanal oximes (*rac*‐2FPPOX, *rac*‐3FPPOX, *rac*‐4FPPOX) were synthesized and purified as racemic *E*‐ and *Z*‐isomers according to previously developed protocols.[^4^, ^22^] The isolated racemic *E*‐ and *Z*‐isomers were subsequently used in biotransformations with *E. coli* whole cells containing the OxdRE in recombinant form. The resulting conversions and enantioselectivities of the formed chiral nitriles are shown in Table 1. The reactions with this wild‐type enzyme proceed as expected, thus furnishing the (*S*)‐nitrile when starting from the *E*‐ isomer whereas utilizing the *Z*‐isomer led to the (*R*)‐nitrile. The determined E‐values and the corresponding calculated energy values of these biotransformation experiments are given in Table 1.

**Table 1:** Biotransformations of various fluoro‐substituted phenyl‐2‐propanal oximes as *E*‐ or Z‐isomers with OxdRE‐WT. 
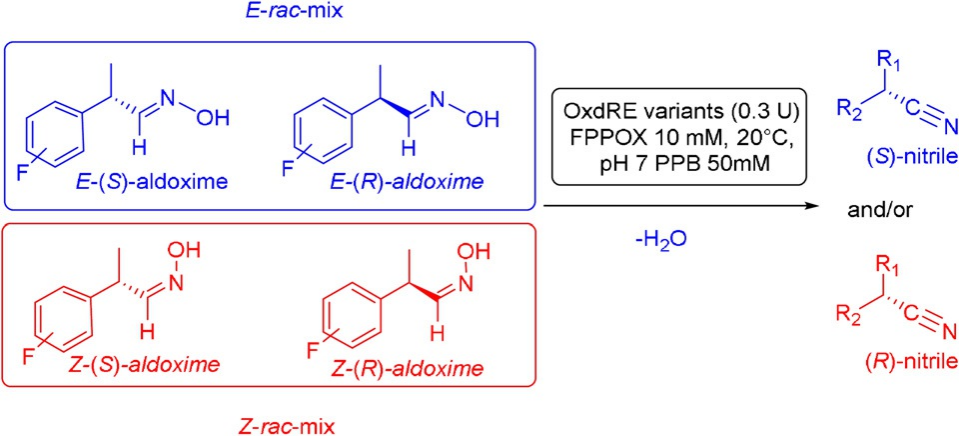

Entry	Name	OxdRE‐WT			
	FPPOX	Conc. [%]	*ee* [%]	E‐value	ΔΔ*G* [kcal mol^−1^]
1	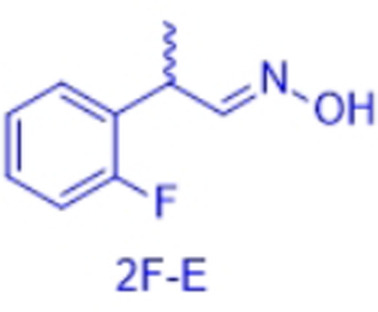	45	96 (*S*)	112	2.6
2	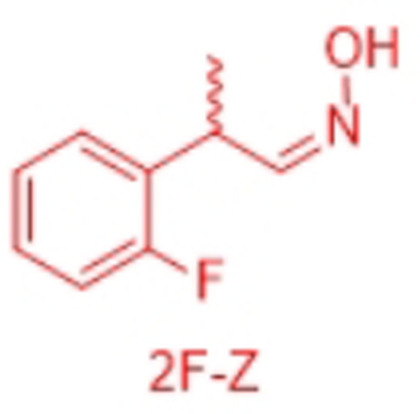	50	65 (*R*)	9	1.2
3	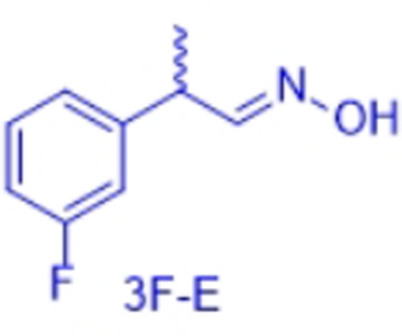	48	96 (*S*)	146	2.7
4	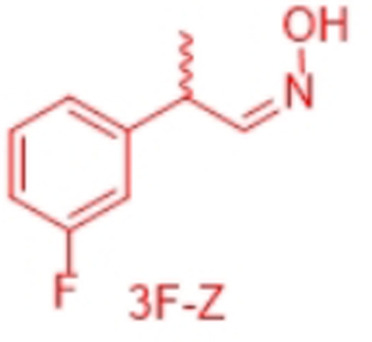	52	64 (*R*)	9	1.2
5	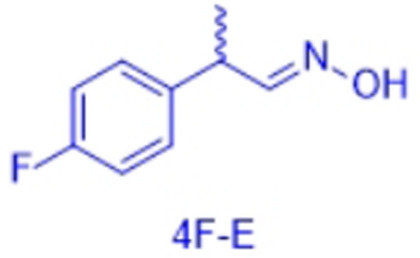	34	93 (*S*)	44	2.1
6	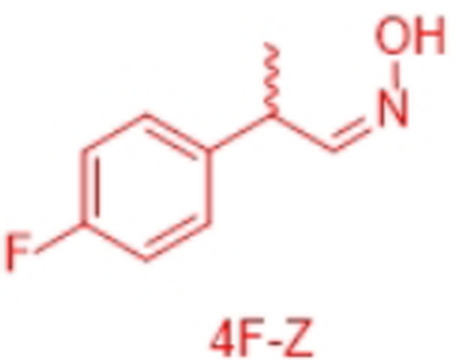	32	54 (*R*)	4	0.7

This aldoxime dehydratase from *Rhodococcus* sp. N‐771 (OxdRE‐WT)^[23]^ has been chosen for our studies since crystal structures are available for this enzyme (PDB 3a15, 3a16, 3a17),^[24]^ which served as an ideal starting point for the molecular modelling of the transition states for the conversion of the various *E*‐ and *Z*‐isomers. In detail, various crystal structures are available, including two structures being co‐crystallized with (non‐chiral) substrates. Furthermore, crystal structures of two different conformations of the OxdRE are known, namely an “open” and “a closed” conformation. Since the available co‐crystals were only found in “closed” conformation, the docking was performed using the closed conformation of OxdRE as this conformation appeared to be the one being relevant for the catalytic cycle.

**Figure 1:**
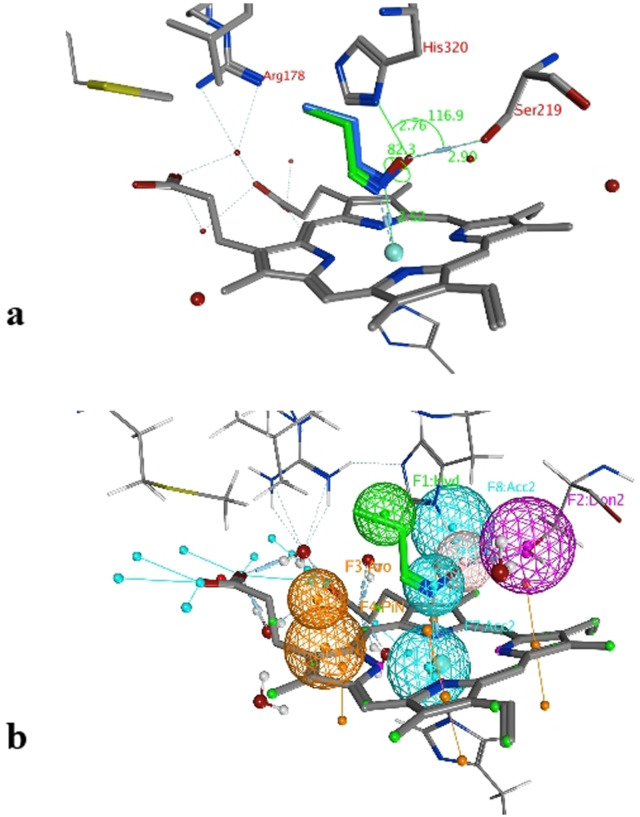
a) Evaluating the docking results with redocking of *n*‐propanal oxime; b) pharmacophore generated with *n*‐propanal oxime.

In order to find out which step in the reaction determines the selectivity, normally all intermediate states must be calculated. As in our case the enantioselectivity of the enzyme depends on the *E*‐ or *Z*‐isomer of the substrate and due to the fact that this *E*/*Z* information only can play a role in the initial step of the catalytic mechanisms during the binding phase of the ligand, we calculated the binding energies of the ligands to rationalize the stereoselectivity of this enzyme class. The MM force field calculation was performed using data generated from preliminary experiments^[8]^ and from this work. To find the correct protein‐ligand‐structures (pose) we used the structure of the co‐crystal^[24]^ with *n*‐propanal oxime as ligand as a basis for our study. Based on the distances and angles of the co‐crystal structure and the mechanism for the dehydration,^[14]^ we determined cut off values for a correct pose (Figure 1 a). With the redocking of the *n*‐propanal oxime we could evaluate and prove our docking model and calculation.

**Figure 2:**
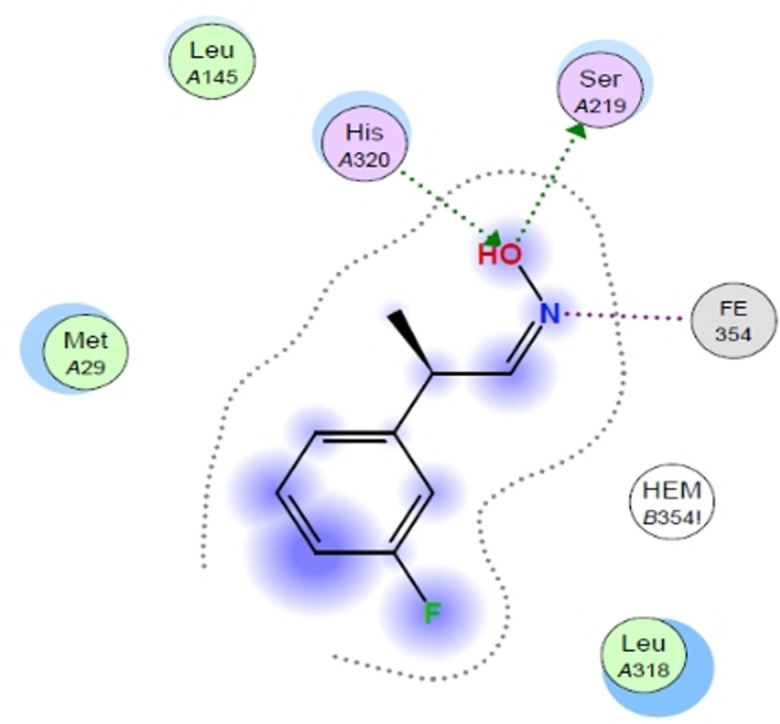
Ligand interaction pattern with (*Z*,*R*)‐3FPPOX as an example bound in the active site of OxdRE‐WT.

To further refine, simplify and automate our docking we used pharmacophores to obtain the correct pose of our ligands (Figure 1 b). The pharmacophore can be used to define the binding motif such as the position of the functional group and its orientation. For example, a good pose is not just described by the presence of the aldoxime ligand inside the pocket, but by the orientation of this ligand, in which the aldoxime function shows a specific position with specified angles (His‐O‐Ser ≈115°), dihedrals (C‐N‐O‐O, ≈85°) and distances (Fe‐N, ≈2.5 Å; O‐Ser, ≈2.8 Å; O‐His, 2.7 Å) towards the heme group and the catalytic triad. Only when these prerequisites are fulfilled, the subsequent dehydration step can proceed. With these defined parameters in hand, a pharmacophore query was created which then allowed us to find ligand poses with this specified binding motif (see Supporting Information). Even though metalloenzymes are not well parameterized[^25^, ^26^] a combination of MMF94 force field and the scoring functions London dG and Affinity dG proved to be suited for this purpose. The protein model was prepared using the preparation kit given by MOE. As a representative example, the 2D‐Figure in Figure 2 shows the interaction pattern of the enantio‐ and diastereomerically pure ligand (*Z*,*R*)‐3FPPOX with the active site of the enzyme OxdRE‐WT.

The ligand shows a hydrogen donation to the Ser219 and the His320 donates a hydrogen towards the oxygen of the ligand, while the nitrogen is coordinated by the Fe^II^‐metal center of the heme. The phenyl group of the ligand interacts with the porphyrin ring via π‐π‐stacking and the aldoxime function for each isomer is always in the same position. It is noteworthy that by means of this modelling tool we were then not only able to predict the enantiopreference, but also to determine quantitatively the enantioselectivity of any aldoxime substrate of this study (Table 2).

**Table 2:** Comparison of docking experiments with OxdRE (3a17) and 2′‐, 3′‐, and 4′‐fluorophenyl‐2‐propanal oximes FPPOX and the experimental biotransformation of OxdRE‐WT with the various aldoximes.^[a]^

Entry	Aldoxime	Δ*G* [kcal mol^−1^]	ΔΔ*G* [kcal mol^−1^]	Pred.	Expt.
1	2F‐*E*,*R*	−7.30	1.87	(*S*)	(*S*)
2	2F‐*E*,*S*	−9.17			
3	2F‐*Z*,*R*	−6.24	1.64	(*R*)	(*R*)
4	2F‐*Z*,*S*	−4.60			
5	3F‐*E*,*R*	−7.68	1.98	(*S*)	(*S*)
6	3F‐*E*,*S*	−9.66			
7	3F‐*Z*,*R*	−6.28	1.51	(*R*)	(*R*)
8	3F‐*Z*,*S*	−4.77			
9	4F‐*E*,*R*	−7.07	2.65	(*S*)	(*S*)
10	4F‐*E*,*S*	−9.72			
11	4F‐*Z*,*R*	−5.88	0.92	(*R*)	(*R*)
12	4F‐*Z*,*S*	−4.96	

[a] Δ*G*: binding energy from docked ligand, ΔΔ*G* difference of binding energies between two enantiomers of one isomer. Lower binding energy of one enantiomer corresponds to the formed enantiomer.

In detail, we calculated the difference (ΔΔ*G*) of the aldoxime ligand binding energies (Δ*G*) in order to predict the enantiopreference and enantioselectivity. For example, the binding energy of (*S*,*E*)‐3F of −9.66 kcal mol^−1^ is lower than the binding energy of the opposite enantiomer (*R*,*E*)‐3F with −7.68 kcal mol^−1^ (Table 2, entries 5, 6). Notably, the resulting calculated energy difference (ΔΔ*G*) of 1.98 kcal mol^−1^ corresponds well with the experimental value of the enantioselectivity and is in agreement with the experimentally observed preferred formation of the *S*‐enantiomer. In general, the ΔΔ*G*‐values were used to determine the enantioselectivity and even though our method is based on force field it is noteworthy that with this type of molecular modelling it was not only possible to rationalize the enantiopreference but also to predict the degree of the enantioselectivity for the investigated substrates in an accurate fashion. The high accuracy is underlined when comparing the ΔΔ*G*‐values from the docking experiments (Table 2) with those obtained from the biotransformation experiments (Table 1) demonstrating a perfect agreement of the predicted enantiopreferences and a good agreement of the quantitative ΔΔ*G*‐values.

**Figure 3:**
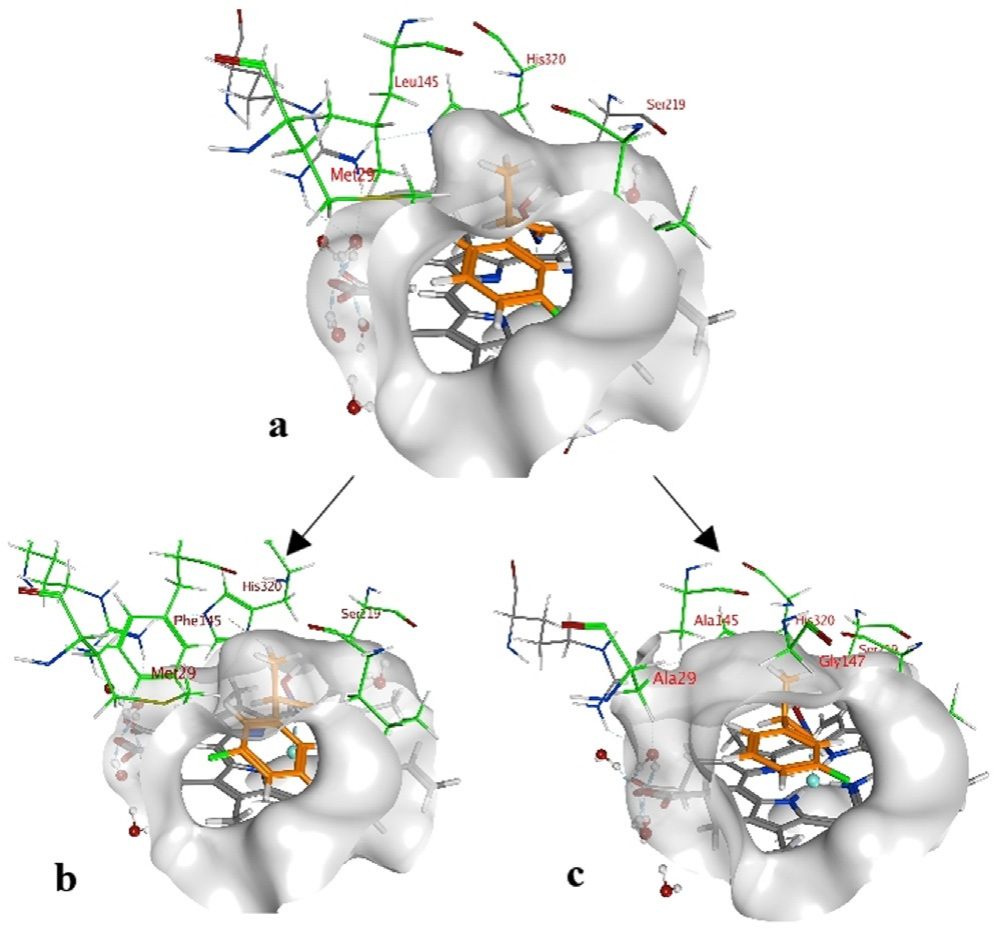
3‐(*Z*,*R*)‐FPPOX bound in the catalytic center of a) OxdRE‐WT, b) OxdRE‐Leu145Phe, and c) OxdRE‐3M. The methyl group is in the cavity, which is formed by the amino acids Met29, Leu145, Ala147, and His320. The figure shows the docked position of the ligand 3‐(*Z*,*R*)‐FPPOX in three different OxdRE variants. The Leu145Phe has a smaller cavity, whereas the triple point mutant 3M (Met29Ala, Leu145Ala, Ala147Gly) has an increased cavity size.

By looking deeper into the final poses of our protein‐ligand‐structures and docked aldoxime ligands we could see similarities between each substrate and its conformers (Figure 3 a). The methyl group is exposed to Met29, Leu145 and the His320. The general pose of the docked ligand does not vary in dependency of the position or the type of the halogen substituent at the phenyl ring. Therefore, the halogen substituent has no direct effect upon the selectivity. However, it has a certain influence on the acceptance of a ligand.

**Figure 4:**
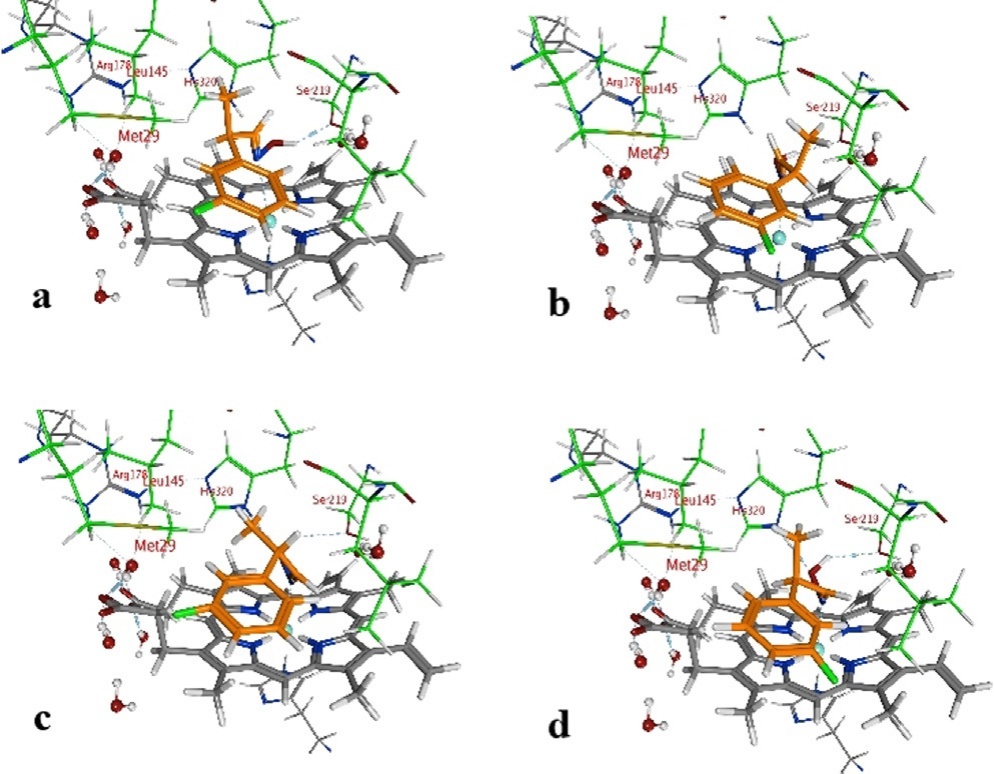
Protein–ligand structure (pose) of 3‐FPPOX conformers in the active center of OxdRE‐WT. a) 3‐(*E*,*R*); b) 3‐(*E*,*S*); c) 3‐(*Z*,*S*); d) 3‐(*Z*,*R*).

The enantiomeric excess can differ because the binding energies vary with the type and position of the substituent at the phenyl ring. The methyl group is the only group which differs among the compared conformers in space and alignment. Based on that information we propose that the arrangement of the methyl group has a major impact on the selectivity for a specific aldoxime isomer. The methyl group is always positioned in a cavity being formed by the amino acids Met29, Leu145, Ala147 and His320, but for each conformer in a specific arrangement (Figure 4).

In general, all PPOX‐derivatives follow a certain alignment. The methyl group of the PPOX‐derivatives is always in the above‐mentioned cavity. However, while the methyl group of the ligands with *E*,*R*‐ and *Z*,*S*‐conformations (not preferred) are closer to the His320 and Leu145, the methyl group of the ligand with *E*,*S*‐ and *Z*,*R*‐conformation (preferred) are more in the middle of the pocket directing towards the cavity (Figure 4). The available data suggest that the position and orientation of the methyl group in relation to the cavity determines the selectivity (Figure 4 a). The necessary alignment is only possible for the *E*‐isomer in *S*‐configuration and for the *Z*‐isomer only in *R*‐configuration. Due to the scoring function, however, it is not possible to determine the single contribution of each van der Waals (VdW) interaction. Thus, a direct determination of the energy contribution of the methyl group was not possible.

Another contribution leading to the observed selectivity could be related to the aldoxime function. Due to its low variation in the alignment, the aldoxime moiety could cause the energy gap (ΔΔ*G*) and consequently the degree of the selectivity. It has been successfully demonstrated, however, that no correlation between energy contribution, selectivity and conformation exists. Therefore, it was concluded that the arrangement of the aldoxime function is essential for the reaction, but insignificant for the differentiation between the enantiomeric pairs of an isomer (see Supporting Information).

In order to prove our hypothesis that the methyl group is the main cause for the selectivity and in order to validate our modelling study further, we decided to calculate and experimentally evaluate various mutants with (i) a more enlarged or alternatively (ii) a tighter cavity in the active site. Accordingly, these mutants then should lead to a decreased (in case of (i)) or increased (in case of (ii)) differentiation of the substrate enantiomers and, thus, to a decrease or increase of the enantioselectivity.

To start with the design of mutants with an enlarged active site (case (i)), our in silico studies showed that mutants with such an increased size of the cavity lead to a significant decrease of the ΔΔ*G*‐value (see Supporting Information). Consequently, such mutants then should lead to a lower selectivity. As described above, the cavity is formed by four amino acids. Leu145 appeared for us to be the most promising position for mutations due to the highest steric contribution to the cavity compared to the amino acids Ala147 and Met29. Thus, replacement of Leu145 with alanine and glycine should lead to a dramatically enhanced cavity. Mutating the proximal His320 to glycine or alanine also would increase the size of the cavity tremendously, but as a part of the catalytic triad it is not possible to mutate His320 without loss of the catalytic activity.^[27]^

**Figure 5:**
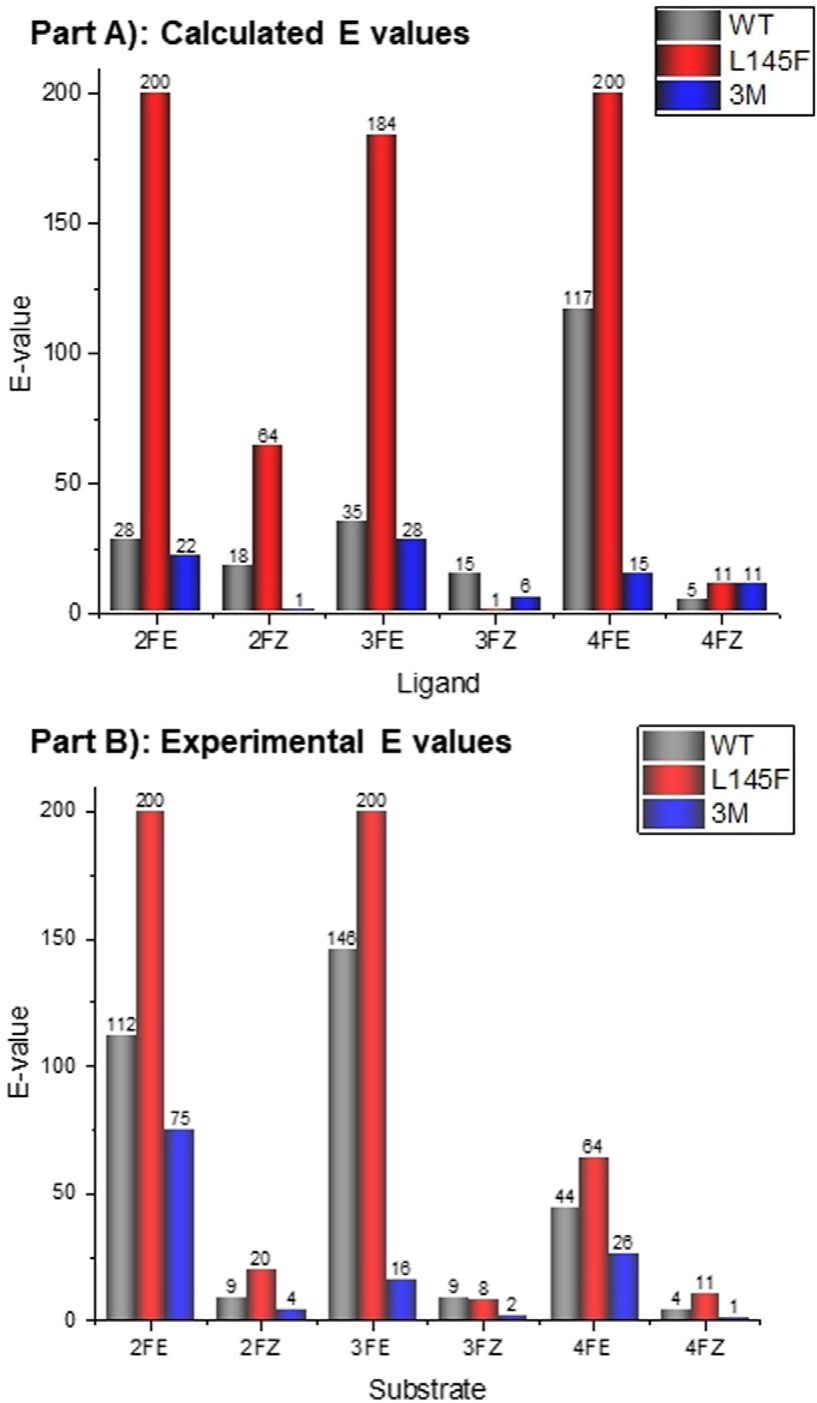
A) Calculated E‐values from the ΔΔ*G* values of the docking data for the FPPOX aldoximes with each enzyme. B) Experimental E‐values determined from the biotransformation of the FPPOX aldoximes to their corresponding nitriles with the use of OxdRE‐WT, OxdRE‐Leu145Phe, and OxdRE‐3 M. The graphs are normalized to a maximum E‐value of 200 (thus, an E value of 200 is graphically shown even when the determined E‐value was >200).

However, the single point mutations of each position did not lead to a significant decrease of the ΔΔ*G*‐value except for the His320 mutation (in silico). Therefore, we designed double and triple point mutations, and the triple point mutation showed a more significant decrease in the ΔΔ*G*‐value. Based on the in silico study, the triple mutant Met29Gly/Leu145Gly/Ala147Gly (OxdRE‐3G) would have represented the most promising mutant. However, in wet experiments this mutant was found to be unstable and the expressed protein was only observed in the insoluble fraction (see Supporting Information). As an alternative, we calculated the mutant Met29Ala/Leu145Ala/Ala147Gly (OxdRE‐3 M; Table 3 and Figure 3 c). This mutant was then also prepared and studied in wet experiments, and the results turned out to be in excellent agreement with our theoretical predictions. These data are shown in Table 3 and Figure 5 and will be discussed in detail below.

**Table 3:** Comparison of docking result for the FPPOX derivatives with OxdRE‐Leu145Phe and OxdRE‐3M. Only isomer pairs with ΔΔ*G* values are shown.

	Name	Leu145Phe		3M	
Entry	PPOX	ΔΔ*G* [kcal mol^−1^]	E^[a]^	ΔΔ*G* [kcal mol^−1^]	E^[a]^
1	2FE	3.65	>200 (*S*)	1.77	22 (*S*)
2	2FZ	2.33	65 (*R*)	0.20	1 (*R*)
3	3FE	2.91	184 (*S*)	1.87	29 (*S*)
4	3FZ	0.06	1 (–)	1.00	6 (*S*)
5	4FE	3.85	>200 (*S*)	1.54	16 (*S*)
6	4FZ	1.36	11 (*R*)	1.40	11 (*S*)

[a] In parentheses, the predicted preferred absolute configuration is given.

As a next step, we focused on a complementary mutation strategy, which now should create enzyme mutants with a decreased size of the cavity (case (ii)), thus making the active site more tight. Accordingly, for the resulting biotransformation then an increased enantioselectivity can be expected. In order to decrease the size of the cavity, the position Leu145 turned out as the only option for a suitable mutation. In contrast, the amino acids Met29 and Ala147 are not suitable for rationally decreasing the size of the cavity. The Ala147 mutations were all in the wrong orientation and not exposed to the active site. Met29 was already one of the longest amino acids, which could reach the cavity. For Leu145 the most promising in silico mutation was found to be Leu145Phe (Figure 3 b). Other amino acids were also tested in silico, but they were either too small, too large or changed the environment of the catalytic triad too much. Our in silico mutation and the performed docking predicted that the Leu145Phe mutant would be a promising candidate for a more enantioselective conversion of PPOX derivatives (Table 3 and Figure 3 b).

With these two optimized mutants in hand (addressing case (i) and (ii)), a detailed theoretical and experimental study was conducted (Table 3, Figure 5). The docking results with the two identified most promising mutants with an increased and decreased cavity, respectively, are shown in Table 3 and Figure 5, Part A). As expected, the ΔΔ*G*‐values are much higher for the Leu145Phe mutant compared to the 3‐M mutant, which is in good agreement with our hypothesis. For example, for the two enantiomers of the 2F*E*‐PPOX substrate, a much higher ΔΔ*G*‐value of 3.65 kcal mol^−1^ was determined in the docking experiment for the OxdRE‐Leu145Phe mutant compared to that for mutant OxdRE‐3M (1.77 kcal mol^−1^) and also compared to the experimentally determined value for the wild‐type enzyme (2.6 kcal mol^−1^) (Table 3, entry 1 and Table 1, entry 1). For a better comparison of the theoretical data with the experimental ones determined from the biotransformations, we calculated the E‐values from the calculated energy values (ΔΔ*G*). Accordingly, in case of 2F*E*‐PPOX these in silico experiments predict an excellent E value for the mutant Leu145Phe with E>200, thus significantly outperforming the wild type enzyme (E=112) as well as the 3M‐mutant (E=22).

In Figure 5, Part A), a comparison of the docking data for all FPPOX derivatives with the three different Oxd enzymes is given. As can be seen in Figure 5, Part A), the Leu145Phe mutant has in general higher E‐values than the wild type, while the 3M‐mutant has lower E‐values. The docking data further predict that in general the enantioselectivity for the conversion of the *Z*‐isomer is lower than the one for the conversion of the *E*‐isomer.

In order to evaluate if these predictions and hypothesis are correct, as next we performed in vitro these mutations of OxdRE and used the mutants in biotransformations (Figure 5, Part B). We were pleased to find that the results from this experimental study proved to be in good agreement with the theoretical data and revealed interesting results in terms of a strongly improved mutant leading to increased enantioselectivities (Figure 5, Part B)). It is noteworthy that in all biotransformations the theoretically predicted tendency of the change of the enantioselectivity (E value) when replacing the wild‐type enzyme by the prioritized mutant Leu145Phe as a biocatalyst was experimentally observed (Figure 5, Parts A and B), thus also confirming the validity of the theoretical study. Furthermore, the improved, high enantioselectivities calculated for the prioritized Leu145Phe‐mutant have been found in the experimental study, leading to excellent E values of >200 in the biotransformations when starting from the aldoxime substrates 2F*E*‐PPOX and 3F*E*‐PPOX (Figure 5, Parts A and B). From a practical point it should be added that the mutant OxdRE‐Leu145Phe, which is still able to convert both *E*‐ and *Z*‐isomers of the aldoxime substrate, shows an increased enantioselectivity in particular for the *E*‐isomer.

Thus, these data also underline the plausibility of our hypothesis of the impact of active site modification on the enantioselectivity. Furthermore, we could demonstrate that based on this rational modelling by means of the in silico approach with the MOE software a more enantioselective aldoxime dehydratase can be generated.

## Conclusion

In conclusion, the stereochemically unique and to the best of our knowledge unprecedented stereochemical phenomena of aldoxime dehydratases, which are able to enantioselectively dehydrate *E*‐ and *Z*‐aldoximes to the opposite enantiomeric forms of a chiral nitrile, has been rationalized by means of a molecular modelling study utilizing an MOE software. This modelling gave a detailed insight why with the same enzyme the use of racemic *E*‐ and *Z*‐aldoximes led to the opposite enantiomeric forms of the chiral nitrile. Furthermore, mutants with an increased and decreased cavity have been calculated, which subsequently showed the expected and theoretically predicted decrease and increase of the enantioselectivities also in the experimental biotransformations. In this connection it was possible to rationally design the mutant OxdRE‐Leu145Phe with a decreased size of the cavity, which then gave superior enantioselectivities compared to the known wild‐type enzyme with excellent E‐values of up to E>200. Thus, this theoretical study can also serve as a validated basis for predicting other aldoxime dehydratase mutants with improved stereochemical properties for novel substrates in the future.

## Conflict of interest

The authors declare no conflict of interest.

## Supporting information

As a service to our authors and readers, this journal provides supporting information supplied by the authors. Such materials are peer reviewed and may be re‐organized for online delivery, but are not copy‐edited or typeset. Technical support issues arising from supporting information (other than missing files) should be addressed to the authors.

Supporting Information
