# Supplementary material for: Rationalizing the Unprecedented Stereochemistry of an Enzymatic Nitrile Synthesis through a Combined Computational and Experimental Approach
Source: Angew Chem Int Ed Engl. 2021 Jul 21;60(35):19162–8. doi: 10.1002/anie.202017234 (PMC8456930; doi:10.1002/anie.202017234)
Supplement: Supplementary file 1 — Supporting Information [file ANIE-60-19162-s001.pdf]

Angewandte Chemie
Eine Zeitschrift der Gesellschaft Deutscher Chemiker

Supporting Information

Rationalizing the Unprecedented Stereochemistry of an Enzymatic Nitrile Synthesis through a Combined Computational and Experimental Approach

*Hilmi Yavuzer, Yasuhisa Asano, and Harald Gröger**

anie_202017234_sm_miscellaneous_information.pdf

SUPPORTING INFORMATION

Table of Content

1 Plasmids 2
2 Microbiological methods 4
3 Aldoxime synthesis 7
4 Modelling with MOE (Molecular Operating Environment) 12
5 Docking results 17
6 Biotransformations 21
7 Analytical data 23
8 References 34

1 Plasmids

1.1 Aldoxime dehydratase from *Rhodococcus erythropolis* (*Rhodococcus* sp. N-771, OxdRE) Base sequence (Accession number: GenBank: AB094201.1)

ATGGGCAGCAGCCATCATCATCATCATCACAGCAGCGGCCTGGTGCCGCGCGGCAGCCATATGGAAAGCGCAATTGGTGAACA
TCTGCAGTGTCCGCGTACCCTGACCCGTCGTGTTCCGGATACCTATACCCCTCCGTTTCCGATGTGGGTTGGTCGTGCAGATGA
TGCACTGCAGCAGGTTGTTATGGGTTATCTGGGTGTTCAGTTTCGTGATGAAGATCAGCGTCCGGCAGCACTGCAGGCAATGCG
TGATATTGTTGCAGGTTTTGATCTGCCGGATGGTCCGGCACATCATGATCTGACCCATCATATTGATAATCAGGGCTATGAAAAC
CTGATTGTGGTGGGTTATTGGAAAGATGTTAGCAGCCAGCATCGTTGGAGCACCAGCACCCCGATTGCAAGTTGGTGGGAAAGC
GAAGATCGTCTGAGTGATGGTCTGGGTTTTTTTCGTGAAATTGTGGCACCGCGTGCAGAACAGTTTGAAACCCTGTATGCATTTC
AAGAAGATCTGCCTGGCGTTGGTGCAGTTATGGATGGTATTAGCGGTGAAATTAACGAACATGGTTATTGGGGTAGCATGCGTG
AACGTTTTCCGATTAGCCAGACCGATTGGATGCAGGCAAGCGGTGAACTGCGTGTTATTGCCGGTGATCCGGCAGTTGGTGGTC
GTGTTGTTGTTCGTGGTCATGATAACATTGCACTGATTCGTAGCGGTCAGGATTGGGCAGATGCCGAAGCAGATGAACGTAGCC
TGTATCTGGATGAAATTCTGCCGACCCTGCAGAGCGGTATGGATTTTCTGCGTGATAATGGTCCTGCAGTTGGTTGTTATAGCAA
TCGTTTTGTGCGCAACATTGATATCGATGGCAATTTTCTGGATCTGAGCTATAACATTGGTCATTGGGCAAGCCTGGATCAGCTG
GAACGTTGGAGCGAAAGCCATCCGACCCATCTGCGTATTTTTACCACCTTTTTTCGCGTTGCAGCCGGTCTGAGCAAACTGCGTC
TGTATCATGAAGTTAGCGTTTTTGATGCAGCAGATCAGCTGTATGAATACATTAATTGTCATCCGGGTACAGGTATGCTGCGTGAT
GCAGTTACCATTGCAGAACATTAA

1.2 Amino acid sequence (Uniprot: Q76K71)

MESAIGEHLQCPRTLTRRVPDTYTPPFPMWVGRADDALQQVVMGYLGVQFRDEDQRPAALQAMRDIVAGFDLPDGPAHHDLTHHID
NQGYENLIVVGYWKDVSSQHRWSTSTPIASWWESEDRLSDGLGFFREIVAPRAEQFETLYAFQEDLPGVGAVMDGISGEINEHGYWG
SMRERFPISQTDWMQASGELRVIAGDPAVGGRVVVRGHDNIALIRSGQDWADAEADERSLYLDEILPTLQSGMDFLRDNGPAVGCYS
NRFVRNIDIDGNFLDLSYNIGHWASLDQLERWSESHPTHLRIFTTFFRVAAGLSKLRLYHEVSVFDAADQLYEYINCHPGTGMLRDAVTI
AEH*

1.3 OxdRE-L145F base sequence

TTAATGTTCTGCAATGGTAACTGCATCACGCAGCATACCTGTACCCGGATGACAATTAATGTATTCATACAGCTGATCTGCTGCAT
CAAAAACGCTAACTTCATGATACAGACGCAGTTTGCTCAGACCGGCTGCAACGCGAAAAAAGGTGGTAAAAATACGCAGATGGG
TCGGATGGCTTTCGCTCCAACGTTCCAGCTGATCCAGGCTTGCCCAATGACCAATGTTATAGCTCAGATCCAGAAAATTGCCATC
GATATCAATGTTGCGCACAAAACGATTGCTATAACAACCAACTGCAGGACCATTATCACGCAGAAAATCCATACCGCTCTGCAGG
GTCGGCAGAATTTCATCCAGATACAGGCTACGTTCATCTGCTTCGGCATCTGCCCAATCCTGACCGCTACGAATCAGTGCAATGT
TATCATGACCACGAACAACAACACGACCACCAACTGCCGGATCACCGGCAATAACACGCAGTTCACCGCTTGCCTGCATCCAAT
CGGTCTGGCTAATCGGAAAACGTTCACGCATGCTACCCCAATAACCATGTTCGTTAATTTCACCGCTAATACCATCCATAACTGCA
CCAACGCCAGGCAGATCTTCTTGAAATGCATAGAAGGTTTCAAACTGTTCTGCACGCGGTGCCACAATTTCACGAAAAAAACCCA
GACCATCACTCAGACGATCTTCGCTTTCCCACCAACTTGCAATCGGGGTGCTGGTGCTCCAACGATGCTGGCTGCTAACATCTTT
CCAATAACCCACCACAATCAGGTTTTCATAGCCCTGATTATCAATATGATGGGTCAGATCATGATGTGCCGGACCATCCGGCAGA
TCAAAACCTGCAACAATATCACGCATTGCCTGCAGTGCTGCCGGACGCTGATCTTCATCACGAAACTGAACACCCAGATAACCCA
TAACAACCTGCTGCAGTGCATCATCTGCACGACCAACCCACATCGGAAACGGAGGGGTATAGGTATCCGGAACACGACGGGTC
AGGGTACGCGGACACTGCAGATGTTCACCAATTGCGCTTTCCAT

1.4 OxdRE-L145F protein sequence

MESAIGEHLQCPRTLTRRVPDTYTPPFPMWVGRADDALQQVVMGYLGVQFRDEDQRPAALQAMRDIVAGFDLPDGPAHHDLTHHID
NQGYENLIVVGYWKDVSSQHRWSTSTPIASWWESEDRLSDGLGFFREIVAPRAEQFETFYAFQEDLPGVGAVMDGISGEINEHGYW
GSMRERFPISQTDWMQASGELRVIAGDPAVGGRVVVRGHDNIALIRSGQDWADAEADERSLYLDEILPTLQSGMDFLRDNGPAVGCY
SNRFVRNIDIDGNFLDLSYNIGHWASLDQLERWSESHPTHLRIFTTFFRVAAGLSKLRLYHEVSVFDAADQLYEYINCHPGTGMLRDAV
TIAEH*

1.5 OxdRE-10-(3M)-M29A-L145A-A147G base sequence

TTAATGTTCTGCAATGGTAACTGCATCACGCAGCATACCTGTACCCGGATGACAATTAATGTATTCATACAGCTGATCTGCTGCAT
CAAAAACGCTAACTTCATGATACAGACGCAGTTTGCTCAGACCGGCTGCAACGCGAAAAAAGGTGGTAAAAATACGCAGATGGG
TCGGATGGCTTTCGCTCCAACGTTCCAGCTGATCCAGGCTTGCCCAATGACCAATGTTATAGCTCAGATCCAGAAAATTGCCATC
GATATCAATGTTGCGCACAAAACGATTGCTATAACAACCAACTGCAGGACCATTATCACGCAGAAAATCCATACCGCTCTGCAGG
GTCGGCAGAATTTCATCCAGATACAGGCTACGTTCATCTGCTTCGGCATCTGCCCAATCCTGACCGCTACGAATCAGTGCAATGT
TATCATGACCACGAACAACAACACGACCACCAACTGCCGGATCACCGGCAATAACACGCAGTTCACCGCTTGCCTGCATCCAAT
CGGTCTGGCTAATCGGAAAACGTTCACGCATGCTACCCCAATAACCATGTTCGTTAATTTCACCGCTAATACCATCCATAACTGCA
CCAACGCCAGGCAGATCTTCTTGAAAGCCATATGCGGTTTCAAACTGTTCTGCACGCGGTGCCACAATTTCACGAAAAAAACCCA
GACCATCACTCAGACGATCTTCGCTTTCCCACCAACTTGCAATCGGGGTGCTGGTGCTCCAACGATGCTGGCTGCTAACATCTTT
CCAATAACCCACCACAATCAGGTTTTCATAGCCCTGATTATCAATATGATGGGTCAGATCATGATGTGCCGGACCATCCGGCAGA
TCAAAACCTGCAACAATATCACGCATTGCCTGCAGTGCTGCCGGACGCTGATCTTCATCACGAAACTGAACACCCAGATAACCCA
TAACAACCTGCTGCAGTGCATCATCTGCACGACCAACCCACGCCGGAAACGGAGGGGTATAGGTATCCGGAACACGACGGGTC
AGGGTACGCGGACACTGCAGATGTTCACCAATTGCGCTTTCCAT

1.6 OxdRE-10-(3M)-M29A-L145A-A147G protein sequence

MESAIGEHLQCPRTLTRRVPDTYTPPFPAWVGRADDALQQVVMGYLGVQFRDEDQRPAALQAMRDIVAGFDLPDGPAHHDLTHHID
NQGYENLIVVGYWKDVSSQHRWSTSTPIASWWESEDRLSDGLGFFREIVAPRAEQFETAYGFQEDLPGVGAVMDGISGEINEHGYW
GSMRERFPISQTDWMQASGELRVIAGDPAVGGRVVVRGHDNIALIRSGQDWADAEADERSLYLDEILPTLQSGMDFLRDNGPAVGCY
SNRFVRNIDIDGNFLDLSYNIGHWASLDQLERWSESHPTHLRIFTTFFRVAAGLSKLRLYHEVSVFDAADQLYEYINCHPGTGMLRDAV
TIAEH*

1.7 OxdRE-25-(3G)-M29G-L145G-A147G base sequence

TTAATGTTCTGCAATGGTAACTGCATCACGCAGCATACCTGTACCCGGATGACAATTAATGTATTCATACAGCTGATCTGCTGCAT
CAAAAACGCTAACTTCATGATACAGACGCAGTTTGCTCAGACCGGCTGCAACGCGAAAAAAGGTGGTAAAAATACGCAGATGGG
TCGGATGGCTTTCGCTCCAACGTTCCAGCTGATCCAGGCTTGCCCAATGACCAATGTTATAGCTCAGATCCAGAAAATTGCCATC
GATATCAATGTTGCGCACAAAACGATTGCTATAACAACCAACTGCAGGACCATTATCACGCAGAAAATCCATACCGCTCTGCAGG
GTCGGCAGAATTTCATCCAGATACAGGCTACGTTCATCTGCTTCGGCATCTGCCCAATCCTGACCGCTACGAATCAGTGCAATGT
TATCATGACCACGAACAACAACACGACCACCAACTGCCGGATCACCGGCAATAACACGCAGTTCACCGCTTGCCTGCATCCAAT
CGGTCTGGCTAATCGGAAAACGTTCACGCATGCTACCCCAATAACCATGTTCGTTAATTTCACCGCTAATACCATCCATAACTGCA
CCAACGCCAGGCAGATCTTCTTGAAAGCCATAGCCGGTTTCAAACTGTTCTGCACGCGGTGCCACAATTTCACGAAAAAAACCCA
GACCATCACTCAGACGATCTTCGCTTTCCCACCAACTTGCAATCGGGGTGCTGGTGCTCCAACGATGCTGGCTGCTAACATCTTT
CCAATAACCCACCACAATCAGGTTTTCATAGCCCTGATTATCAATATGATGGGTCAGATCATGATGTGCCGGACCATCCGGCAGA
TCAAAACCTGCAACAATATCACGCATTGCCTGCAGTGCTGCCGGACGCTGATCTTCATCACGAAACTGAACACCCAGATAACCCA
TAACAACCTGCTGCAGTGCATCATCTGCACGACCAACCCACCCCGGAAACGGAGGGGTATAGGTATCCGGAACACGACGGGTC
AGGGTACGCGGACACTGCAGATGTTCACCAATTGCGCTTTCCAT

1.8 OxdRE-10-(3M)-M29G-L145G-A147G protein sequence

MESAIGEHLQCPRTLTRRVPDTYTPPFPGWVGRADDALQQVVMGYLGVQFRDEDQRPAALQAMRDIVAGFDLPDGPAHHDLTHHID
NQGYENLIVVGYWKDVSSQHRWSTSTPIASWWESEDRLSDGLGFFREIVAPRAEQFETGYGFQEDLPGVGAVMDGISGEINEHGYW
GSMRERFPISQTDWMQASGELRVIAGDPAVGGRVVVRGHDNIALIRSGQDWADAEADERSLYLDEILPTLQSGMDFLRDNGPAVGCY
SNRFVRNIDIDGNFLDLSYNIGHWASLDQLERWSESHPTHLRIFTTFFRVAAGLSKLRLYHEVSVFDAADQLYEYINCHPGTGMLRDAV
TIAEH*

2 Microbiological methods

2.1 Site directed mutagenesis via Quick-Change PCR

To construct OxdRE-mutants with increased or decreased cavity size, the cavity forming amino acids were changed by means of site directed mutagenesis. As method the Quick-Change PCR was used. In Table 1 the components for the Quick-Change PCR are listed.

Table 1: Component mixture for PCR-Reaction:

Component	Volume/µL
HF-Buffer	5
dNTP (10 mM)	0.5
Fw-Primer (10 µM)	0.5
Rw-Primer (10 µM)	0.5
Phusion-Polymerase (0.02 U/µL)	0.5
Template (50 ng/µL)	2.0
DMSO	1
dH_2O	15.0

The designed primers are listed in Table 2. As example Met29Ala_fw and Met29Ala_rw are the forward and reverse primers for the mutation of the Methionine29 towards Alanine29. The same goes for the Leu147Ala_fw, Leu147Ala_rw, Ala145Gly_fw and Ala145Gly_rw. Also, the mutated sequences are displayed in red.

Table 2: Designed primers for the Quick-Change PCR.

Primer	Sequence
Met29Ala_fw	CCTCCGTTTCCGGCGTGGGTTGGTCGT
Met29Ala_rw	ACGACCAACCCACGCCGGAAACGGAGG
Met29Gly_fw	CTCCGTTTCCGGGGTGGGTTGGTCGTGCA
Met29Gly_rw	TGCACGACCAACCCACCCCGGAAACGGAG
Leu147Ala_fw	ACAGTT TGAAACCGCGTATGCATTTCAAGAAG
Leu147Ala_rw	CTTCTTGAAATG CATACGCGGTTTCAAACTGT
Ala145Gly_fw	TGAAATCCATACAGGGTTTCAA
Ala145Gly_rw	TGAAACCCTGTATGGATTTCAAG
Leu318Gly_fw	CGCTAACTTCATGATAGGCACGCAGTTTGC
Leu318Gly_rw	GCAAACTGCGTGCCTATCATGAAGTTAGCG
Leu145Gly_fw	AGTTTGAAACCGGCTATGCATTTCAAGAAGAT
Leu145Gly_rw	ATCTTCTTGAAATGCATAGCCGGTTTCAAACT
Leu145Gly/ Ala147Gly_fw	AGTTTGAAACCGGCTATGGCTTTCAAGAAGAT
Leu145Gly/Ala 147Gly_rw	ATCTTCTTGAAAGCCATAGCCGGTTTCAAACT
Leu145Ala/ Ala147Gly_fw	ATCTTCTTGAAAGCCATATGCGGTTTCAAACT
Leu145Ala/ Ala147Gly_rw	AGTTTGAAACCGCATATGGCTTTCAAGAAGAT
OxdRE_Leu145Phe_fw	AGAACAGTTTGAAACCTTCTATGCATTTCAAGA A
OxdRE_Leu145Phe_rw	TTC TTG AAATGATAGAAGGTTTCAAA CTGTTC T

The Quick-Change PCR was done with the following program in Table 3.

Table 3: QC-PCR-program.

PCR-Steps	Temperature/°C	Time/s	Cycle
Initial denaturation	98	120	1
Denaturation	98	30	25
Annealing	58-64	30s	25
Elongation	72	64	25
Final elongation	72		1
Storage	4	∞	1

2.2 DPN1 -Treatment, Ligation and Transformation

The PCR-product was treated with *DPN1* for 1 hour at 37 degrees. After the *DPN1* treatment the PCR-product was treated with T4-ligase, which is not a necessary step, but recommended one to ligate the PCR-product to a closed vector. This closed vector was then transformed to competent *Bl21 DE3* (Star) cells.

For the transformation the competent cells, which are stored at -80°C, are placed in a vessel with ice. Then the ligation solution or the plasmid of choice is added to the competent cells and incubated for 30 minutes. Afterwards the cells are heat shock treated for 90 seconds at 42°C. Then 700 µL LB-Medium is added and the cell solution is incubated for 3 hours at 37°C and 1200 rpm. The suspension was then centrifuged at 11.000 g and 600 µL of LB-Medium was discarded. The cell pellet was then resuspended again in the left 100 µL medium. The cell suspension of 100 µL was then poured over an LB-agar-plate and incubated over night at 37°C

2.2 Agar plate preparation

The agar plates were prepared by dissolving 10 g of LB-medium, 6 g of agar in 400 mL of water. The solution is then sterilized using an autoclave. The thick solution of agar is cooled down until ~60°C, before adding the respective antibiotics (100 µg/ml endconentration). Afterwards the solution is poured into sterile plates.

2.3 Preculture

The preliminary cultures were prepared in test tubes. 5 ml of LB-medium and the desired antibiotic with a concentration of 50 µg/ ml were transferred to the sterile test tubes. The colonies were removed from the plate with a toothpick and then used to inoculate the precultures.

2.4 Main culture

For the main culture, sterilized 500 mL-Flask with LB-Medium (400 mL) and Kanamycin (50 $\mu g \cdot mL^{-1}$) were used. The cultivating started by adding 5 ml of the preculture. The main culture was incubated at 20°C, 155 rpm for 72 hours. The so-called leaky expression was the most suited one for the expression of aldoxime dehydratase.

Sodium dodecyl sulfate polyacrylamide gel electrophoresis (SDS-Page)

Table 4: Solutions and components for SDS-PAGE

Solution	Composition
Collector gel (4%)	2.8 mL dH_2O
	1.3 mL 0.5 M Tris-HCl (pH 6.8)
	50.0 µL SDS (10%)
	0.8 mL Acrylamide
	50.0 µL APS
	5.0 µL TEMED
Separation gel (15%)	3.5 mL dH_2O
	4.0 mL acrylamide (33%)
	2.5 mL,1.5 M Tris-HCl (pH 8.8)
	100.0 µL SDS (10%)
	100.0 µL APS (10%)
	10.0 µL TEMED

The gel was prepared according to Table 4 and loaded with 10 µL of 1 mg/mL protein solutions. The protein concentrations were measured via standard Bradford assay, using Bradford reagent and protocol from Sigma-Aldrich. The samples were mixed with 6 µL of loading buffer and filled the rest with water to a total volume of 30 µL and an end concentration of 1 mg/ml. A voltage of 80 V was set for the collecting gel. When the separation gel was reached, the voltage was increased to 120 V, the maximum current reached was 15 mA. The stained gel shown in figure 1.

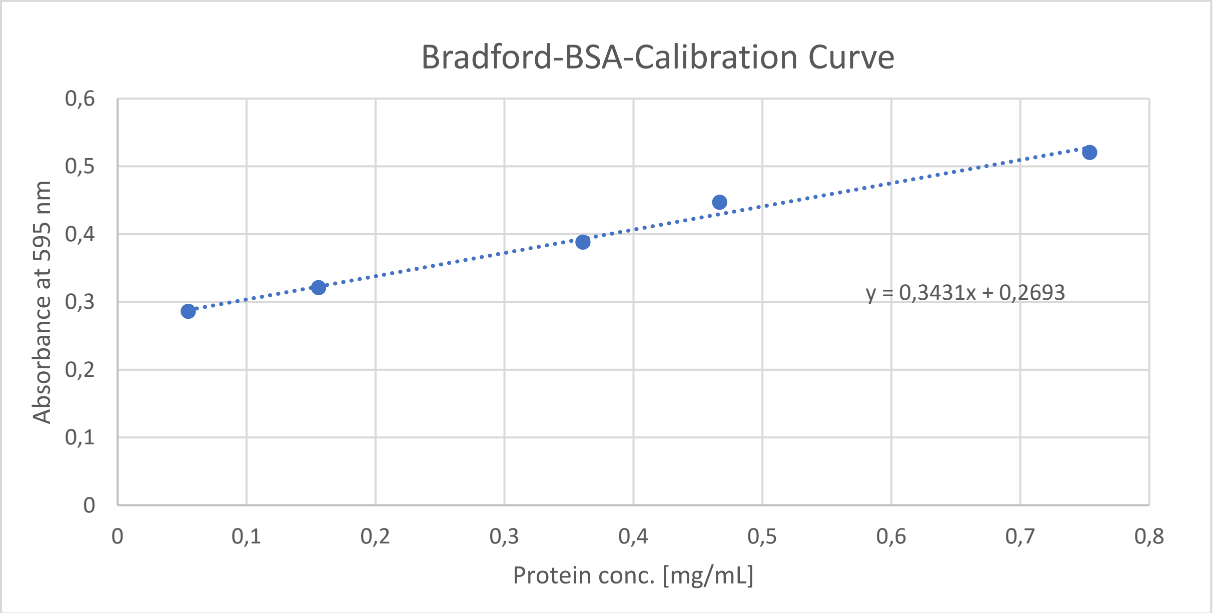

Table 5: The protein concentration of the SDS samples determined via BSA standard curve. The samples were standardized to a concentration of 1mg/mL and applied on the SDS gel.

Sample	Protein conc./mg/mL	Sample volume/ µL	Leamli buffer/ µL	Water/ µL	total volume/ uL
OxdRE-WT	6,3	4,8	6,0	19,2	30
OxdRE-L145F	3,9	7,8	6,0	16,2	30
OxdRE-3M	3,1	9,8	6,0	14,2	30
OxdRE-3G	3,2	9,4	6,0	14,6	30
OxdRE-Wt-lys	8,7	3,4	6,0	20,6	30
OxdRE-L145F-lys	5,6	5,4	6,0	18,6	30
OxdRE-3M-lys	4,1	7,3	6,0	16,7	30
OxdRE-3G-lys	6,7	4,5	6,0	19,5	30

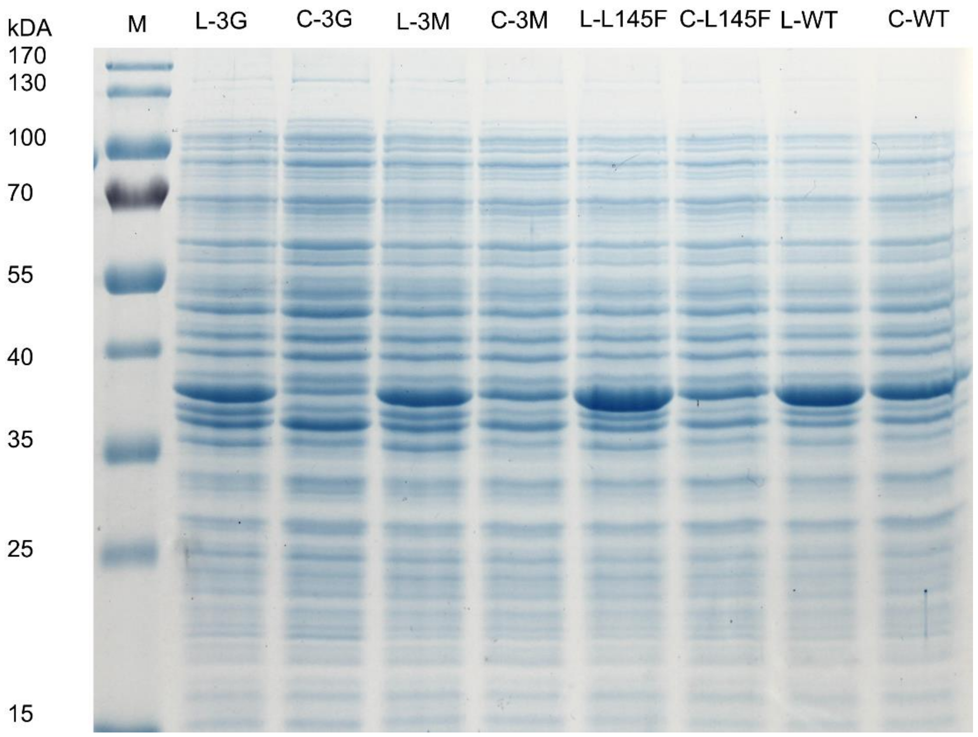

Figure 1: SDS-PAGE of OxdRE-WT, OxdRE-L145F, OxdRE-3M and OxdRE-3G. C indicated crude extract while L indicates lysate. The SDS-PAGE show the over expression of the Oxds in general by the means of lysate solution with the comparison to the crude extract.

The SDS-PAGE shows that the mutants are as well expressed as the wild type OxdRE, but they are not as stable as the wild type, with much smaller bands in the crude extract as in the lysate. For example, the OxdRE-3G-mutant is not stable at all with no protein band on the SDS-PAGE for the crude extract row.

3 Aldoxime synthesis

3.1 General procedure 1 (GP 1): nitroaldol condensation of fluoro-benzaldehyde (1) with nitromethane

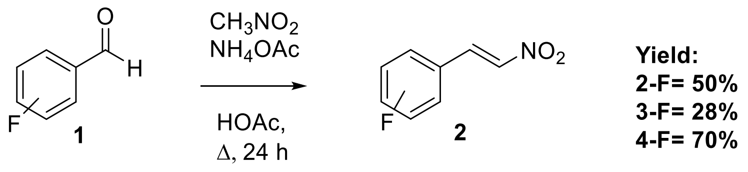

The synthesis was carried out in analogy to Betke et al.[1] Fluoro-benzaldehyde (1.0 eq.), ammonium acetate (0.1 eq.) and nitromethane (6.5 eq.) were dissolved in acetic acid. The solution was heated to reflux for 24 hours. The complete conversion was determined by means of TLC (cyclohexane/ethyl acetate, 6:1, v/v). Water (1:1, v/v) was then added and the phases separated. The solid was filtered off and recrystallized from ethanol.

Synthesis of 2-fluoro-(*E*)-(2-nitrovinyl)-benzene (2a)

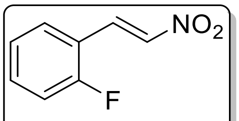

2a
$C_8H_6FNO_2$
Mw: 167,14 g/mol

The synthesis was carried out according to GP1. 2-fluoro-benzaldehyde (10.0 g, 80.0 mmol, 8.55 mL) was dissolved in acetic acid (30 mL) with nitromethane 21 mL, 400 mmol) and ammonium acetate (500 mg, 6.49 mmol) and heated to reflux. After workup, the product was obtained as an orange solid.

Yield: 7.6 g, 57 %.

^{1}H NMR (500 MHz, Chloroform-*d*) δ [ppm]: 8.08 (d, ^3J = 13.7 Hz, 1H, C*H*=CH), 7.76 (d, ^3J = 13.8 Hz, 1H, C*H*=CH), 7.58 – 7.48 (m, 2H, Ar-H), 7.30 – 7.25 (m, 1H, Ar-*H*), 7.21 (dd, ^3J = 10.8, 8.3 Hz, 1H, Ar-*H*).

The ^{1}H-NMR-spectroscopy were compared with an analog compound from literature[1]

Synthesis of 3-fluoro-(*E*)-(2-nitrovinyl)-benzene (2b)

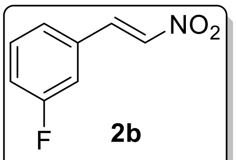

2b
$C_8H_6FNO_2$
Mw: 167.14 g/mol

The synthesis was carried out according to GP1. 3-fluorobenzaldehyde (20.0 g, 161 mmol) was dissolved in acetic acid (30 mL) with nitromethane (40 mL, 800 mmol) and ammonium acetate (500 mg, 6.49 mmol) and heated to reflux. After workup, the product was obtained as a yellow solid.

Yield: 7.6 g, 28%.

^{1}H NMR (500 MHz, Chloroform-*d*) δ [ppm]: 7.97 (d, ^3J = 13.7 Hz, 1H, CH=C*H*), 7.56 (d, ^3J = 13.7 Hz, 1H, CH=C*H*), 7.44 (td, ^3J = 8.1, 6.1 Hz, 1H, Ar-H), 7.34 (d, *J* = 7.6 Hz, 1H, Ar-H), 7.21 (ddd, *J* = 16.7, 7.9, 2.3 Hz, 2H, Ar-H)

The ^{1}H-NMR-spectroscopy were compared with an analog compound from literature[1]

Synthesis of 4--fluoro-(*E*) -(2-nitrovinyl)-benzene

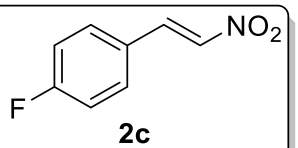

2c
$C_8H_6FNO_2$
Mw: 167.14 g/mol

The synthesis was carried out according to GP1. 4-fluorobenzaldehyde (10.0 g, 80 mmol, 8.55 mL) was dissolved in acetic acid (30 mL) with nitromethane (21 mL, 400 mmol) and ammonium acetate (500 mg, 6.49 mmol) and heated to reflux. After workup, the product was obtained as a yellow solid.

Yield: 8.85 g, 66 %.

^{1}H NMR (500 MHz, Chloroform-*d*): δ [ppm] 7.98 (d, *J* = 13.6 Hz, 1H, CH=C*H*), 7.58 – 7.51 (m, 3H, Ar-H, CH=C*H*), 7.19 – 7.12 (m, 2H, Ar-H).

The ^{1}H-NMR-spectroscopy were compared with an analog compound from literature[1]

General procedure 2 (GP 2): Michael-addition of methyl-magnesium-bromide to fluoro-nitrovinyl benzene

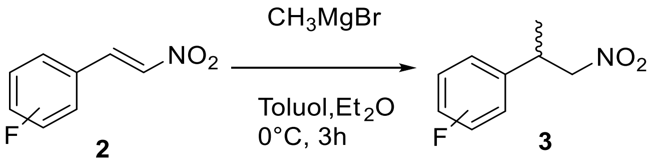

Yield:
2-F= 41%
3-F= 51%
4-F= 52%

In analogy to *Betke et al*[1], the synthesis was carried out in a heat dried flask under argon atmosphere. Dry toluene was placed in the flask and cooled to 0 ° C. After addition of the fluoro-nitrovinyl benzene (1 eq), a 3 M solution of methyl magnesium bromide in diethyl ether (1.5 eq) was added dropwise under argon reflux and further stirring. After three hours of reaction time at 0 ° C., a conversion control was conducted by means of TLC (cyclohexane/ethyl acetate, 6:1, v/v). The reaction mixture was mixed with a saturated ammonium chloride solution (1:1, v/v). The phases were separated, and the aqueous phase was extracted three times with ethyl acetate (1:1, v/v). The combined organic phase was washed with saturated sodium chloride solution (1:3, v/v). The organic phase was dried over magnesium sulfate and the solvent was then evaporated *in vacuo*. The crude product was then purified by automated column chromatography (cyclohexane/ethyl acetate 6:1, v/v) to give the racemic nitroalkane as oil.

Synthesis of *rac*-2-fluoro-1-(1-nitropropan-2-yl) benzene (3a)

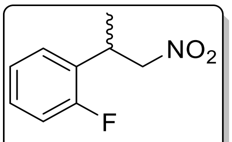

3a

$C_9H_{10}FNO_2$

Mw: 183.18 g/mol

The synthesis of the *rac*-2-fluoro-1-(1-nitropropan-2-yl) benzene was carried out according to GP 2. 2-fluoro-(*E*)-(2-nitrovinyl) benzene (7.30 g, 43.3 mmol) was dissolved in toluene (150 mL) and then methyl magnesium bromide (15 mL, 45 mmol) was added dropwise. After work up, a pink oil was obtained.

Yield: 4.32 g, 53%.

^{1}H NMR (500 MHz, Chloroform-*d*) δ [ppm]: 7.29 – 7.20 (m, 1H, Ar-H), 7.12 (td, *J* = 7.6, 1.3 Hz, 1H, Ar-H), 7.06 (dd ^3J = 10.7, 8.3 Hz, 1H, Ar-H), 4.66 (dd, ^3J = 12.3, 7.0 Hz, 1H, C*H$_2$*), 4.55 (dd, ^3J = 12.3, 8.1 Hz, 1H, C*H$_2$*), 3.89 (h, *J* = 7.3 Hz, 1H, C*H*-CH$_3$), 1.41 (d, ^3J = 7.1 Hz, 3H, C*H$_3$*).

The ^{1}H-NMR-spectroscopy were compared with an analog compound from literature[1]

Synthesis of *rac*-3-fluoro-1-(1-nitropropan-2-yl) benzene (3b)

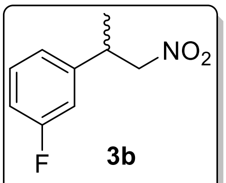

$C_9H_{10}FNO_2$

Mw: 183.18 g/mol

The synthesis of the *rac*-3-fluoro-1-(1-nitropropan-2-yl) benzene was carried out according to GP 2. 3-fluoro-(*E*)-(2-nitrovinyl) benzene (7.3 g, 42.6 mmol) was dissolved in toluene (150 mL) and methyl magnesium bromide (22.6 mL, 68 mmol) was then added dropwise. After work up, an orange oil was obtained

Yield: 0.44 g, 51 %.

^{1}H NMR (500 MHz, Chloroform-*d*) δ [ppm]: 7.24 – 7.15 (m, 2H, Ar-H), 7.03 (dd, ^3J = 8.6, 7.0 Hz, 2H, Ar-H), 4.57 – 4.40 (m, 2H, C*H$_2$*), 3.63 (h, ^3J = 7.3 Hz, 1H, C*H*-CH$_3$), 1.37 (dd, ^3J = 7.0, 1.2 Hz, 3H, C*H$_3$*).

The ^{1}H-NMR-spectroscopy were compared with an analog compound from literature[1]

Synthesis of *rac*-4-fluoro-1- (1-nitropropan-2-yl) benzene (3c)

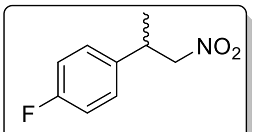

3c

$C_9H_{10}FNO_2$

Mw: 183.18 g/mol

The synthesis of the *rac*-4-fluoro-1-(1-nitropropan-2-yl) benzene was carried out according to GP 2. 4-fluoro-(*E*)-(2-nitrovinyl) benzene (15.1 g, 101.7 mmol) was dissolved in toluene (150 mL) and methyl magnesium bromide (51 mL, 152 mmol) was then added dropwise. After work up, a yellow oil was obtainedn

Yield: 9.7 g, 52%.

^{1}H NMR (500 MHz, Chloroform-*d*) δ [ppm]: 7.24 – 7.15 (m, 2H, Ar-H), 7.03 (dd, ^3J = 8.6, 7.0, 1.9 Hz, 2H), 4.57 – 4.40 (m, 2H, CH$_2$), 3.63 (h, ^3J = 7.3 Hz, 1H, C*H*-CH$_3$), 1.37 (dd, ^3J = 7.0, 1.2 Hz, 3H, C*H*-CH$_3$).

The ^{1}H-NMR-spectroscopy were compared with an analog compound from literature[1]

General procedure 3 (GP 3): Synthesis of *rac*-(*E/Z*)-fluoro-phenylpropanal oxime using benzyl bromide

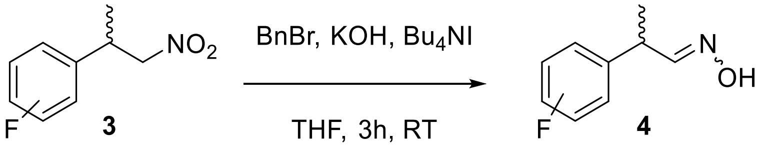

Yield:
2-F= 56%
3-F= 52%
4-F= 38%

The synthesis was carried out in a heat dried flask under argon atmosphere. Dry THF was KOH (85 wt.% pellets, 1.05 eq.) was dissolved in dry THF, 4 Å molecular sieve were added, and the suspension was stirred for half an hour. Benzyl bromide (1.1 eq.) and tetrabutylammonium iodide (TBAI, 0.05 eq.) were added. The respective nitroalkane was then slowly added, dropwise. The suspension was stirred at room temperature for three hours. The conversion was controlled by means of TLC. Water (1:1 v/v) was added and the phases were separated. The aqueous phase was extracted three times with ethyl acetate. The combined organic phases were washed with brine and dried over magnesium sulfate. The solvent was removed under reduced pressure and the crude product obtained as an oil. *Rac*-(*E/Z*)-fluoro-phenylpropanal oximes were purified via automated column chromatography using the biotage system (cyclohexane/ethyl acetate), the mixture of *E*- and *Z*-isomers were obtained as colorless solid or as an oil. The aggregate state was dependent on the *E*/*Z* ratios.

Synthesis of the *rac*-(*E/Z*)-2-fluoro-2-phenylpropanal oximes (4a)

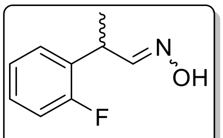

4a

$C_9H_{10}FNO$

Mw: 167.18 g/mol

Synthesis of the *rac*-(*E/Z*)-2-fluoro-phenyl-2-propanal oxime was performed according to GP 3. Potassium hydroxide pellets (1.3 g, 25.0 mmol),4 Å molecular sieve (1.0 g) and THF (20 mL) were placed in the reaction flask (100 mL), then the mixture was stirred at RT for half an hour. Subsequently, benzyl bromide (2.5 mL, 21.0 mmol) and TBAI (360 mg, 1.1 mmol) were added to the reaction mixture and finally the rac-2-fluoro-1- (1-nitropropan-2-yl) benzene (4.2 g , 23.0 mmol) was added dropwise. The suspension was stirred at RT for three hours. Workup by means of automated chromatography (cyclohexane/ethyl acetate 6/1, v/v) led to a mixture of the *E*- and *Z*-diastereomers.

Yield: 2.1 g, 56%. *E/Z*-ratio 80/20

^{1}H NMR (500 MHz, DMSO-d_6) δ [ppm]: 10.94 (s, 1H, *Z*-NO*H*), 10.63 (s, 1H, *E*--NO*H*), 7.47 (d, ^3J = 5.7 Hz, 1H, *E*-C*H*=NOH), 7.43 – 7.07 (m, 7H, Ar-H), 6.86 (d, 1H, *Z*-C*H*=NOH), 4.44 (p, ^3J = 7.1 Hz, 1H, *E*-C*H*-CH_3), 3.89 (p, ^3J = 6.9 Hz, 1H, *E*-C*H*-CH_3), 3.33 (s, 1H, DMSO), 1.31 (d, ^3J = 7.2 Hz, 3H, C*H*$_3$).

^{13}C NMR (500 MHz, DMSO-d_6) δ [ppm]: 163.01, 161.07, 134.33, 129.45, 122.65, 116.30, 29.60, 21.07

IR [cm^{-1}]: 3243 (ʋ, O-H), 2967 (ʋ, Ar-H), 2888 (ʋ, CH_3), 1594 (ʋ, C=C), 1489-1444 (δ, C-C-H), 1220 (ʋ, C-F).

HRMS: (ESI, positive ions): calculated for $C_9H_{10}FNOH^+$: m/z 168.077 [M+H]$^+$, found 168.082

Synthesis of the *rac*-(*E/Z*)-3-fluoro-2-phenylpropanal oximes

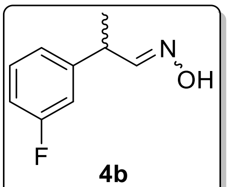

4b

$C_9H_{10}FNO$

Mw: 167.18 g/mol

The synthesis of the *rac* (*E/Z*)-3-fluoro-2-phenylpropanal oxime was carried out according to GP3. THF (20 mL) were introduced into the reaction vessel, and potassium hydroxide pellets were added to 85% (1.2 g, 24.0 mmol) and 4 Å molecular sieve (4 g) and the mixture was stirred for half an hour at room temperature. Subsequently, benzyl bromide (3.5 mL, 28.8 mmol) and TBAI (300 mg, 1.0 mmol) were added to the reaction mixture, and finally the rac-3-fluoro-1-(1-nitropropan-2-yl) benzene (3.9 g , 20.8 mmol) was added dropwise. The suspension was stirred at RT for three hours. Workup by means of and automated chromatography (cyclohexane/ethyl acetate 6/1, v/v) led to a mixture of the *E*- and *Z*-diastereomer.

Yield: 1.9g, 52 %, *E/Z*-ratio: 71/29

^{1}H NMR (500 MHz, DMSO-d_6) δ [ppm]: 10.95 (s, 1H, *Z*-NO*H*), 10.6 (s, 1H, *E*-NO*H*), 7.41 (d, ^3J = 6.2 Hz, 1H, *E*-C*H*=NOH), 7.40 – 7.33 (m, 1H, Ar-H), 7.16 – 7.01 (m, 3H, Ar-H), 6.81 (d,^3J= 7.1 Hz, 1H, *Z*-C*H*=NOH), 3.65 (p, *J* = 7.1 Hz, 1H, *E*-C*H*-CH_3), 1.35 (d, *J* = 7.1 Hz, 3H, C*H*$_3$).

^{13}C NMR (500 MHz, DMSO-d_6) δ [ppm]: 163.30, 161.37, 152.09, 146.02, 130.48, 123.45, 114.09 , 113.34, 19.15

^{19}F NMR (500 MHz, DMSO-d6) δ [ppm]: -113.13,-113.28 (without standard)

IR [cm^{-1}]: 3239 (ʋ, O-H), 2971 (ʋ, Ar-H), 2888 (ʋ, CH_3), 1616-1607 (ʋ, C=C), 1462-1439 (δ, C-C-H),1284-1242 (ʋ, C-F).

HRMS: (ESI, positive ions): calculated for $C_9H_{10}FNOH^+$: m/z 168.077 [M+H]$^+$, found 168.082

Synthesis of the *rac*-(*E/Z*)-4-fluoro-2-phenylpropanal oximes

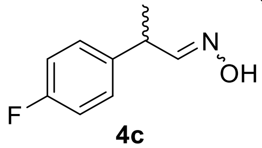

4c

$C_9H_{10}FNO$

Mw: 167.18 g/mol

Synthesis of the *rac* (*E/Z*)-4-fluoro-phenyl-2-propanal oxime was performed according to GP3.THF (80 mL) were introduced into the reaction vessel, and then potassium hydroxide pellets were added, 85% (2.9 g, 20.8 mmol) and 4 Å molecular sieve (8 g), and the mixture was stirred at RT for half an hour. Subsequently, benzylbromide (6.2 mL, 0.5 mmol) and TBAI (900 mg, 2.4 mmol) were added to the reaction mixture, and finally, the *rac*-4-fluoro-1-(1-nitropropan-2-yl) benzene (9.5 g , 51 mmol) was added dropwise thereto. The suspension was stirred at RT for three hours. Workup by means of automated chromatography (cyclohexane/ethyl acetate 6/1, v/v) led to a mixture of the *E*- and *Z*-diastereomers.

Yield: 3.4 g, 40%, *E/Z*-ratio: 69/31

^{1}H NMR (500 MHz, DMSO-d_6) δ [ppm]: 10.91 (s, 1H, *Z*-NO*H*), 10.57 (s, 3H, *E*-NO*H*), 7.42 (d, *J* = 6.1 Hz, 4H, *E*-C*H*=NOH), 7.30 (dd, *J* = 19.3, 8.4, 5.6 Hz, 2H, Ar-H), 7.19 – 7.06 (m, 2H, Ar-H), 6.78 (d, *J* = 7.2 Hz, 1H, *Z*-C*H*=NOH), 4.19 (p, ^3J = 7.2 Hz, 1H, *E*-C*H*-CH_3) 3.65 (p, *J* = 6.9 Hz, 1H, *Z*-C*H*-CH_3), 1.33 (d, *J* = 7.1 Hz, 3H, C*H*$_3$).

The identity was confirmed via ^{1}H-NMR-spectroscopy.[1]

General procedure 4 (GP 4): Copper catalyzed nitrile synthesis

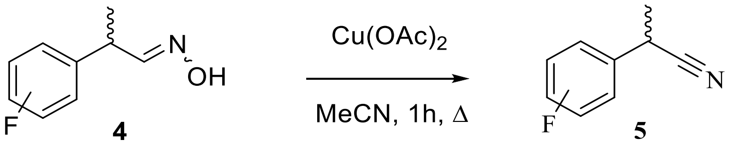

Copper(II)acetate (0.1 eq.) was dissolved in acetonitrile at RT. *Rac*-(*E/Z*)-fluoro-phenylpropanal oximes (1.0 eq.) were added and heated to reflux for 90 minutes. Conversion was controlled by means of TLC (cyclohexane/ethyl acetate, 6: 1 v/v). The solvent was removed under reduced pressure to give the crude product. The crude product was dissolved in cyclohexane/ethyl acetate (8:1, v/v) and purified by means of a glass frit silica gel (silica gel, 3 cm filling height). The pure product was obtained as an oil.[1]

Synthesis of the *rac*-2-(2-fluorophenyl)-propanenitrile

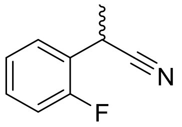

5a
C_9H_8FN
Mw: 149.17 g/mol

The synthesis of the *rac*-2-(2-fluorophenyl)-propanenitrile was carried out according to GP4. Copper(II)acetate (8.2 mg, 45 µmol) was dissolved in 5 mL acetonitrile. The aldoxime (**7a**) (116.0 mg, 694.0 µmol) was then added. Workup by column chromatography (cyclohexane/ethyl acetate, 8:1, v/v) led to the product as a colorless oil.
Yield: 65 mg, 63%
^{1}H NMR (500 MHz, DMSO-d_6) δ [ppm]: 7.50 (td, ^3J = 7.7, 1.5 Hz, 1H, Ar-H), 7.47 – 7.39 (m, 1H, Ar-H), 7.32 – 7.22 (m, 2H, Ar-H), 4.46 (q, ^3J = 7.2 Hz, 1H, C*H*-CH_3), 1.54 (dd, ^3J = 11.2, 7.2 Hz, 3H, C*H*$_3$).
^{13}C NMR (500 MHz, DMSO-d_6) δ [ppm]:
IR [cm^{-1}]: 2999 (υ, Ar-H), 2933 (υ, CH_3), 2248 (υ, C=N) , 1603 (υ, C=C), 1509 (δ, C-C-H), 1201 (υ, C-F)
MS: (ESI, positive ions): calculated for $C_9H_8FNH^+$: m/z 299.1 [2M+H]$^+$, found 301.2

Synthesis of the *rac*-2-(3-fluorophenyl)-propanenitrile

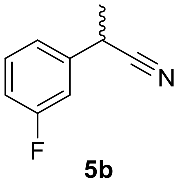

5b
C_9H_8FN
Mw: 149.17 g/mol

The synthesis of the *rac*-2-(3-fluorophenyl)-propanenitrile was carried out according to GP4. Copper(II)acetate (8.2 mg, 45 µmol) was dissolved in 5 mL acetonitrile. The aldoxime (60.0 mg, 359 µmol) was then added. After working up by column chromatography (cyclohexane/ethyl acetate, 8:1, v/v), product was obtained as colorless oil.

Yield: 50 mg, 92%
^{1}H NMR (500 MHz, DMSO-d_6) δ [ppm]: 7.52 – 7.42 (m, 1H, Ar-H), 7.28 (dd, ^3J = 9.2, 2.7 Hz, 2H, Ar-H), 7.23 – 7.16 (m, 1H, Ar-H), 4.35 (q, ^3J = 7.2 Hz, 1H, C*H*-CH_3), 1.55 (dd,^3J = 7.3, 1.2 Hz, 3H, C*H*$_3$).
^{13}C NMR (500 MHz, DMSO-d_6) δ [ppm]:
IR [cm^{-1}]: 2999 (υ, Ar-H), 2939 (υ, CH_3), 2242 (υ, C=N),1609-1600 (υ, C=C), 1484-1444 (δ, C-C-H), 1284-1239 (υ, C-F).
MS: (ESI, positive ions): calculated for $C_9H_8FNH^+$: m/z 299.1 [2M+H]$^+$, found 301.2

Synthesis of the *rac*-2-(4-fluorophenyl)-propanenitrile

The synthesis of the *rac*-2-(4-fluorophenyl)-propanenitrile was carried out according to GP4. Copper(II)acetate (8.2 mg, 45 µmol) was dissolved in 5 mL acetonitrile. The aldoxime (71.0 mg, 452.0 µmol) was then added. After working up by column chromatography (cyclohexane/ethyl acetate, 8:1, v/v), the product was obtained as a colorless oil.

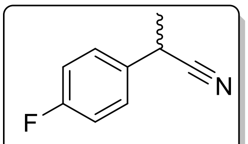

5c
C_9H_8FN
Mw: 149.17 g/mol

Yield: 60 mg, 88%
^{1}H NMR (500 MHz, DMSO-d_6) δ [ppm]: 7.46 (dd, *J* = 8.3, 5.3 Hz, 2H, Ar-H), 7.25 (t, *J* = 8.6 Hz, 2H, Ar-H), 4.32 (q, *J* = 7.2 Hz, 1H, C*H*-CH_3), 1.53 (d, *J* = 7.2 Hz, 3H, C*H*$_3$).
The identity was confirmed via ^{1}H-NMR-spectroscopy.[1]

4 Modelling with MOE (Molecular Operating Environment)

4.1 General procedure for docking with MOE

The docking was performed with MOE (Molecular Operating Environment).[2] First the protein was prepared, therefore the co-crystal of OxdRE (PDB 3A16) was used. The preparation included protonation, adding missing atoms, preparing the heme structure and minimizing the protein energy in the corresponding force-field (MMFF94). The ligands were prepared using MOE to design the ligands and minimize within the force-field of interest. Over many optimization steps regarding the placement and scoring, including also reaction mechanism and experimental data, a final docking method was developed to find realistic protein ligand structures. These protein ligand structures (pose) were then used for general rationalizing of the enzyme's selectivity. In the following part procedure for a successful docking is tailored.

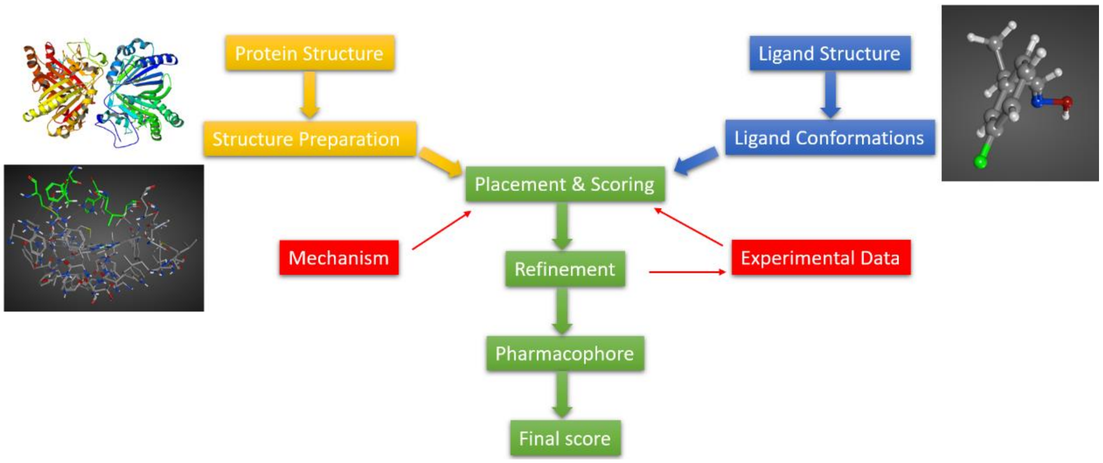

4.2 Initial docking:

The PDB data 3A16 contained the crystal structure of OxdRE as co-crystal with propanal oxime as ligand was loaded into the software MOE (molecular operating environment). Using the co-crystal and the postulated mechanism for aldoxime dehydratase we could tailor the binding motif of the ligand. According to the mechanism the aldoxime has to bind the heme iron(II) and need to be coordinated via serine and histidine. The serine is here the hydrogen acceptor and the histidine the hydrogen donor. The co-crystal does only show the serine and the heme interaction towards the aldoxime as ligand (Scheme 1). Even though the hydrogen donation towards the aldoxime is not recognized, the oxygen π- electrons show towards the histidine, which indicates the right ligand binding. Furthermore, the binding motif itself was then quantified in binding distances, angles and dihedrals. Afterwards the same ligand (propanal oxime) was redocked into the active site. The redocked structure gave us the variance of these quantified values, which we then could use as "cut-off" values for other ligands (Scheme 2).

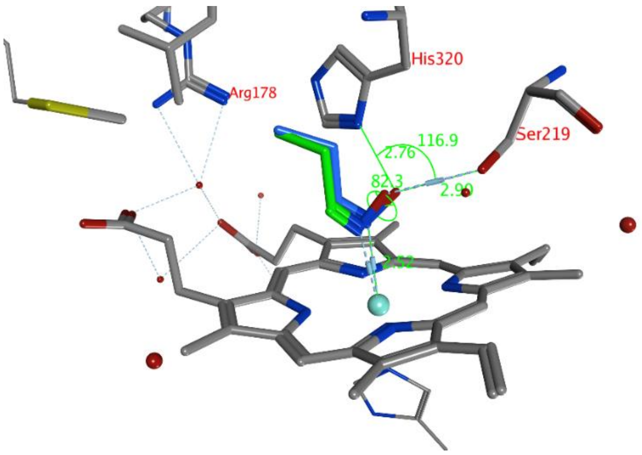

Scheme 1: Propanal oxime as Co-crystal and docked structure in OxdRE-WT. Showing distances, angles and dihedrals of the correct pose. Green ligand (docked) blue ligand (co-cystal).

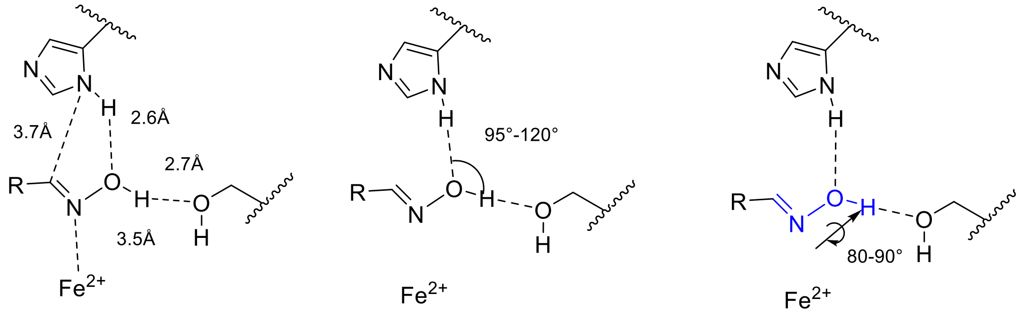

Scheme 2: Schematic representation of the bond angles, distances and dihedrals as 2D structure.

4.2.1 Docking with ligands of interest phenyl propanal oxime derivatives

The enzyme can convert PPOX derivatives into opposite enantiomers depending on the isomer used for the reaction. First of all, for each substrate with two enantiomers and two isomers we generated in total 4 ligands for the docking study. The ligands were then docked inside the active site using GDP 1 (general docking procedure 1). The results gave us a hint how the ligand could fit inside the pocket (active site) of the enzyme. The final poses for each ligand did not show any interaction patterns in terms of hydrogen bonds or metal ligation. Also the amount of possible poses are way too many and different, which does not allow any comparison between each ligand.

4.2.2 Placement:

Triangle matcher: Poses are generated by aligning ligand triplets of atoms on triplets of alpha spheres in a more systematic way than in the Alpha Triangle method.

Pharmacophore: Information from the pharmacophore is directly used to place the ligand in the binding site. This is the preferred method when a pharmacophore is present, but will not work if there is no pharmacophore.

4.3 Pharmacophore generation

The pharmacophore docking describes a placement method where the ligand is forced into specified areas for indicated interaction patterns, such as hydrogen bonds metal ligation and hydrophobic or aromatic interaction. The pharmacophore docking is more powerful when a lead structure and a mechanism is already known. In this case, the co-crystal as the lead structure, as well as the mechanism is already known and given[3]. In figure 2 the generated pharmacophore is shown.

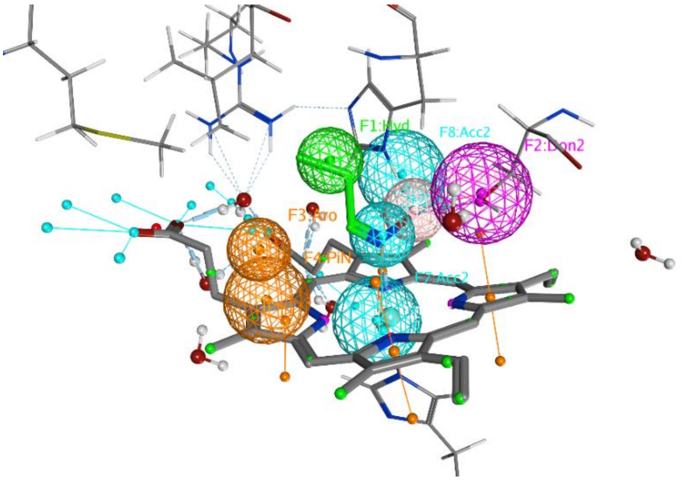

Figure 1: Pharmacophore generated with propanal oxime

The orange sphere describes an aromatic interaction and the green sphere a hydrophobic interaction. Both of these pharmacophores are not essential just giving a possible position for either an aromatic or a hydrophobic part of the ligand. The nitrogen is a hydrogen acceptor (blue sphere) with the right angle the electron pair need to show in the direction of the iron(II), which is why the

bigger blue sphere (hydrogen acceptor projection) is around the iron(II). The hydroxyl group is hydrogen acceptor as well as a donator. Therefore, the sphere for the hydroxyl group has also two different projection spheres one for hydrogen acceptor projection (towards histidine) and one for hydrogen donator projection (towards serine). This projection spheres are created according to the co crystal and the mechanism behind the aldoxime dehydration. The position and the projections are determined with the measured angles, dihedrals and distances. The pharmacophore as such is the result of the correct binding distances, angles and dihedrals.

The pharmacophore can be used to define the binding motif such as the position of the functional group and its orientation. For example, a good pose is not just be described by the presence of the ligand inside the pocket but by the orientation of the aldoxime ligand, in which the aldoxime function shows a specific position with specified angles (His-O-Ser ~115°), dihedrals (C-N-O-O, ~85°) and distances (Fe-N, ~2.5 Å; O-Ser, ~2.8 Å; O-His, 2.7 Å) towards the heme group and the catalytic triad. Only when these prerequisites are fulfilled, the subsequent dehydration step can proceed. With these defined parameters in hand, a pharmacophore query was created which then allowed us to find ligand poses with this specified binding motif. Even though metalloenzymes are not well parameterized[4.5] a combination of MMF94 force field and the scoring functions London dG and Affinity dG proved to be suited for this issue. The protein model was prepared using the preparation kit given by MOE. As a representative example, the 2D-figure in Figure 2 shows the interaction pattern of the enantio- and diastereomerically pure ligand (*Z*,*R*)-3FPPOX with the active site of the enzyme OxdRE-WT. The ligand shows a hydrogen donation to the Ser219 and the His320 donates a hydrogen towards the oxygen of the ligand, while the nitrogen is coordinated by the Fe^{II}-metal center of the heme. The phenyl group of the ligand interacts with the porphyrin ring *via* π-π-stacking and the aldoxime function for each isomer is always in the same position.

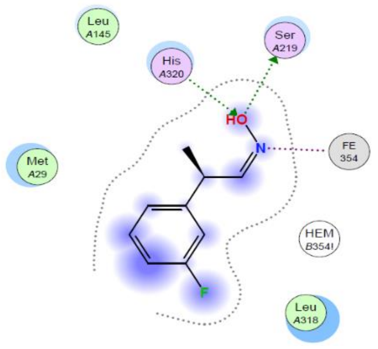

Figure 2: Ligand interaction pattern with (*Z*,*R*)-3FPPOX as example bound in the active site of OxdRE-WT

4.4 Set up and parameters

Forcefield and parameter set up. MMFF94 is well parametrized for nitrogen interactions. Docking was performed with the MMF94 force field. In the following the potential set up with MOE is shown (left side). Enabled are all potential energy terms for bonded, van der Waals and electrostatic interaction and restraints. The cut off for non-bonded interaction with 8 to 10 was not changed. For solvation we used the reaction field model. The threads value just indicates the number of processors which were used for the calculation. In addition, in the following the docking panel in MOE is also shown (right side).

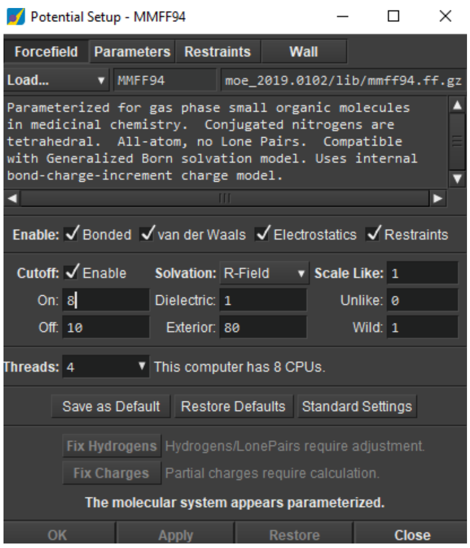

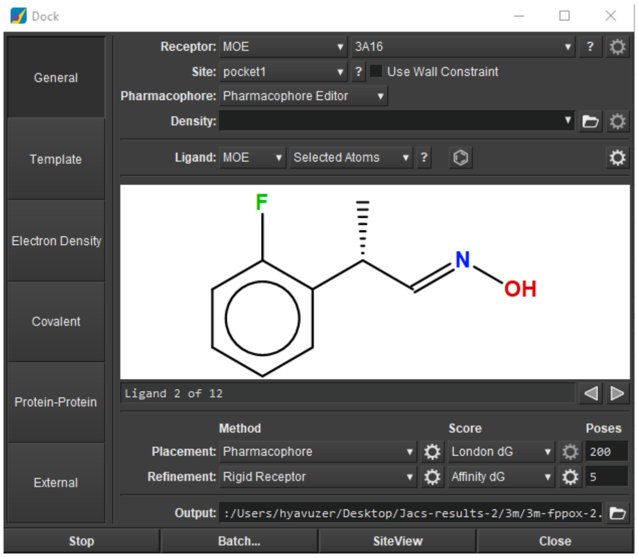

Affinity dG Scoring: This function estimates the enthalpic contribution to the free energy of binding using a linear function:

$$G = C_{hb} f_{hb} + C_{ion} f_{ion} + C_{mlig} f_{mlig} + C_{hh} f_{hh} + C_{hp} f_{hp} + C_{aa} f_{aa}$$

where the f terms fractionally count atomic contacts of specific types and the C's are coefficients that weight the term contributions to the affinity estimate. The individual terms are[2]:

Term	Description
hb	Interactions between hydrogen bond donor-acceptor pairs. An optimistic view is taken; for example, two hydroxyl groups are assumed to interact in the most favorable way.
ion	Ionic interactions. A Coulomb-like term is used to evaluate the interactions between charged groups. This can contribute to or detract from binding affinity.
mlig	Metal ligation. Interactions between Nitrogens/Sulfurs and transition metals are assumed to be metal ligation interactions.
hh	Hydrophobic interactions, for example, between alkane carbons; these interactions are generally favorable.
hp	Interactions between hydrophobic and polar atoms; these interactions are generally unfavorable.
aa	An interaction between any two atoms. This interaction is weak and generally favorable.

London dG Scoring (default): The London dG scoring function estimates the free energy of binding of the ligand from a given pose. The functional form is a sum of terms:

$$\Delta G = c + E_{flex} + \sum_{h-bonds} c_{HB} f_{HB} + \sum_{m-lig} c_M f_M + \sum_{atoms\ i} \Delta D_i$$

where c represents the average gain/loss of rotational and translational entropy; E_{flex} is the energy due to the loss of flexibility of the ligand (calculated from ligand topology only); f_{HB} measures geometric imperfections of hydrogen bonds and takes a value in [0,1]; c_{HB} is the energy of an ideal hydrogen bond; f_M measures geometric imperfections of metal ligations and takes a value in [0,1]; c_M is the energy of an ideal metal ligation; and Di is the desolvation energy of atom i. The difference in desolvation energies is calculated according to the formula

$$\Delta D_i = c_i R_i^3 \left\{ \iiint_{u \notin A \cup B} |u|^{-6}\, du - \iiint_{u \notin B} |u|^{-6}\, du \right\}$$

where A and B are the protein and/or ligand volumes with atom i belonging to volume B; R_i is the solvation radius of atom *i* (taken as the OPLS-AA van der Waals sigma parameter plus 0.5 Angstrom); and c_i is the desolvation coefficient of atom *i*. The coefficients {c, c_{HB}, c_M, c_i} were fitted from ~400 x-ray crystal structures of protein-ligand complexes with available experimental pK_i data. Atoms are categorized into ~12 atom types for the assignment of the c_i coefficients. The triple integrals are approximated using Generalized Born integral formulas.[2]

Energy adjustment

After finding the right placement method, the scoring of these poses had to be adjusted, since only the structures, which had the essential interactions should be in the comparison data file and the energy values should represent the experimental data. The weighting of the parameters for hydrogen bond, metal ligation, hydrophobic contact and hydrophobic-polar interaction were increased. The adjustment in parameters led to improved protein-ligand-structure and also the associated energy values. These values and structures would then describe the best approximation of reality.

	Energy scoring											
#	Atom-atom	Hydrophob-polar	Hydrop.-contact	Metal-ligation	Ionic interaction	Hydrog.-bond						
							ER	ES	ZR	ZS	diff E	diff Z
1	-0.00834	0.02497	-0.01235	-1.0	1.0	-1.0	7.13	7.42	6.49	6.07	-0.29	0.42
2	-0.00834	0.02497	-0.01235	-1.0	1.0	-2.0	10.3	10.85	8.82	8.03	-0.55	0.79
3	-0.00834	0.02497	-0.01235	-1.0	1.5	-2.0	10.3	10.85	8.82	8.03	-0.55	0.79
4	-0.00834	0.02497	-0.01235	-2.0	1.0	-2.0	10.49	11.23	9.55	8.64	-0.74	0.91
5	-0.00834	0.02497	-0.0247	-2.0	1.0	-2.0	14.1	14.81	13.12	12.22	-0.71	0.9
6	-0.00834	0.05	-0.01235	-2.0	1.0	-2.0	5.92	6.75	5.36	4.31	-0.83	1.05
7	-0.02	0.05	-0.01235	-2.0	1.0	-2.0	13.49	14.2	12.58	11.61	-0.71	0.97
8	-0.00834	0.05	-0.01235	-3.0	1.0	-3.0	9.29	10.56	8.41	6.83	-1.27	1.58
9	-0.00834	0.05	-0.01235	-3.5	1.0	-3.5	10.97	12.46	9.94	8.81	-1.49	1.13
10	-0.00834	0.06	-0.01235	-3.5	1.0	-3.5	9.14	10.67	8.27	6.44	-1.53	1.83
11	-0.00834	0.07	-0.01235	-3.5	1.0	-3.5	7.32	8.89	6.6	4.47	-1.57	2.13
12	-0.00834	0.07	-0.0247	-3.5	1.0	-3.5	10.5	12.47	9.49	8.25	-1.97	1.24

General docking procedure 1

Receptor is set to 3A16 including the protein, the solvent and the heme as receptor. Docking site was the complete active site including all amino acids next to the heme group. No walls of constraints were used. Pharmacophore was set to none. No electron density file. The ligand was set to MOE selected atoms, which includes all selected ligands for the calculation. As placement the triangle matcher and as refinement the rigid receptor option was used. The scoring functions for initial score was London dG and for final score affinity dG. Number of placement poses were set to 200 and the number of shown poses to 20.

General docking procedure 2

Receptor is set to 3A16 including the protein the solvent and the heme as receptor. Docking site was the complete active site including all amino acids next to the heme group. No walls of constraints were used. The designed pharmacophore file was chosen for the pharmacophore set up. No electron density file. The ligand was set to MOE selected atoms, which includes all selected ligands for the calculation. As placement the pharmacophore search were chosen and as refinement the rigid receptor option was used. The scoring functions for initial score was London dG and for final score affinity dG. Number of placement poses were set to 200 and the number of shown poses to 5.

5 Docking results

5.1 Docking results with wild type OxdRE

Docking of PPOX derivatives for OxdRE-WT was performed according to GDP2. Also, the partial energy contribution of the aldoxime function was determined.

Table 6: Docking results with OxdRE-WT and all mono substituted chloro-, bromo- and fluoro- PPOX derivatives (as for the ΔΔG-values, the absolute values are given (without sign); for each specific *E*- or *Z*-aldoxime, these values were calculated from the ΔG-values of the two related enantiomers of this aldoxime).

#	*PPOX*	*ΔG/ kcal · mol-1*	*ΔΔG/ kcal · mol^{-1}*	*Enantiomer*	*OH-Ser/ kcal · mol^{-1}*	*Fe-N/ kcal · mol^{-1}*	*His-OH/ kcal · mol^{-1}*	*Sum-energies/ kcal · mol^{-1}*	*Δ-sum energies/ kcal · mol^{-1}*
1	ER	-7.22	2.15	*S*	-2.4	-0.7		-3.1	-0.6
2	ES	-9.37			-0.5	-2		-2.5	
3	ZR	-5.92	1.23	*R*	-0.6	-2.4	-1.8	-4.8	-0.6
4	ZS	-4.69			-0.5	-2	-1.7	-4.2	
5	2FER	-7.30	1.87	*S*	-2.4	-0.7		-3.1	-0.6
6	2FES	-9.17			-0.5	-2		-2.5	
7	2FZR	-6.24	1.64	*R*	-0.9	-2.6	-2	-5.5	-1.2
8	2FZS	-4.60			-0.6	-2	-1.7	-4.3	
9	3FER	-7.68	1.98	*S*	-2.5	-0.5		-3	-0.5
10	3FES	-9.66			-0.5	-2		-2.5	
11	3FZR	-6.28	1.51	*R*	-0.7	-2.3	-1.7	-4.7	-0.4
12	3FZS	-4.77			-0.6	-2	-1.7	-4.3	
13	4FER	-7.07	2.65	*S*	-2.7	-0.9		-3.6	-0.9
14	4FES	-9.72			-1.3	-1.4		-2.7	
15	4FZR	-5.88	0.92	*R*	-0.6	-2.4	-1.8	-4.8	-0.5
16	4FZS	-4.96			-0.6	-2	-1.7	-4.3	
17	2BrER	-7.18	1.01	*S*	-2.6	-1		-3.6	0.2
18	2BrES	-8.19			-2.8	-1		-3.8	
19	2BrZR	-8.26	0.73	*R*	-1.6	-2.4	-1.7	-5.7	-1.5
20	2BrZS	-7.53			-0.7	-2.3	-1.2	-4.2	
21	3BrER	-6.06	2.41	*S*	-2.1	-0.8		-2.9	0.6
22	3BrES	-8.47			-2.8	-0.7		-3.5	
23	3BrZR	-7.00	0.75	*S*	-1.3	-2.6	-0.5	-4.4	1.2
24	3BrZS	-7.75			-1.6	-2.4	-1.6	-5.6	
25	4BrER	-5.20	4.31	*S*	-1.4	-1.1		-2.5	0
26	4BrES	-9.51			-0.5	-2		-2.5	
27	4BrZR	-6.18	0.84	*R*	-0.7	-2.4	-1.8	-4.9	-0.4
28	4BrZS	-5.34			-0.7	-2.1	-1.7	-4.5	
29	2ClER	-6.47	1.55	*S*	-2.7	-1.2		-3.9	-0.9
30	2ClES	-8.02			-2.4	-0.6		-3	
31	2ClZR	-7.78	2.89	*R*	-1.4	-2.5	-1.6	-5.5	-3.5
32	2ClZS	-4.89				-2		-2	
33	3ClER	-7.48	2.32	*S*	-2.5	-0.8		-3.3	-0.7
34	3ClES	-9.80			-1.3	-1.3		-2.6	
35	3ClZR	-10.05	5.04	*R*	-1.1	-2.4		-3.5	1
36	3ClZS	-5.01			-0.7	-1.7	-2.1	-4.5	
37	4ClER	-5.27	4.79	*S*	-2	-1.5		-3.5	-0.4
38	4ClES	-10.06			-1	-2.1		-3.1	
39	4ClZR	-6.36	1.20	*R*	-0.8	-2.4	-1.8	-5	-0.8
40	4ClZS	-5.16			-0.6	-1.6	-2	-4.2	

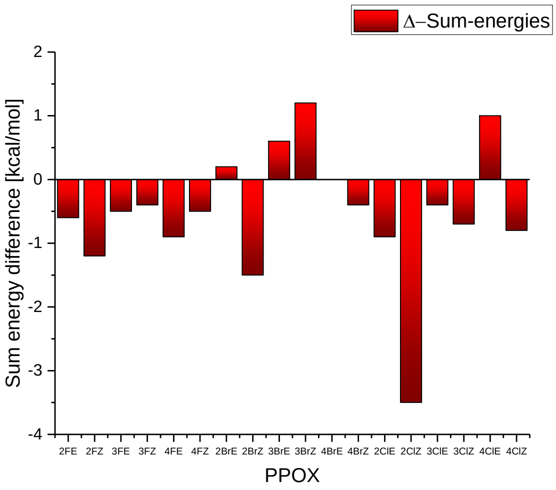

Figure 3: Energy contribution of the aldoxime function in dependency of the enantiomer formed.

The energy contribution of the aldoxime function is random (Figure 3) there is no dependency between the enantioselectivity and the interaction pattern of the functional group. Figure 3 shows the sum of energy contributions of the functional group in dependency of the formed enantiomer. For example, when taking the value obtained from the ΔG-value for the S-enantiomer of a specific *E*- or *Z*-aldoxime minus the ΔG-value for the *R*-enantiomer of this *E*- or *Z*-aldoxime, 2FE has a ΔΔG-value of 1.87 kcal mol^{-1}, which is positive and predicts the *S*-enantiomer (2FER-2FES, -7.36 kcal mol^{-1}-(-9.31 kcal · mol^{-1})), therefore the Δ-sum-energy has to be also positive, but in fact it is not. A dependency with the enantioselectivity would be indicated with an alternating graph.

Docking results with OxdRE-L145F

Docking of PPOX derivatives for the L145F mutant of OxdRE was performed according to GDP2. The partial energy contribution was not determined. Red numbers indicate low docking quality.

Table 7: Docking results with OxdRE-L145F and all mono substituted chloro-, bromo- and fluoro-PPOX derivatives (as for the ΔΔG-values, the absolute values are given (without sign); for each specific *E*- or *Z*-aldoxime, these values were calculated from the ΔG-values of the two related enantiomers of this aldoxime).

#	PPOX	ΔG/ kcal · mol^{-1}	ΔΔG/ kcal · mol^{-1}	Enantiomer
1	2FER	-7.20	3.65	*S*
2	2FES	-10.85		
3	2FZR	-8.74	2.33	*R*
4	2FZS	-6.41		
5	3FER	-7.52	2.91	*S*
6	3FES	-10.43		
7	3FZR	-5.69	0.06	-
8	3FZS	-5.75		
9	4FER	-7.19	3.85	*S*
10	4FES	-11.04		
11	4FZR	-7.84	1.36	*R*
12	4FZS	-6.48		
13	2BrER	-7.32	1.43	*S*
14	2BrES	-8.75		
15	2BrZR	-7.47	0.12	*R*
16	2BrZS	-7.35		
17	3BrER	-7.49	3.73	*S*
18	3BrES	-11.22		
19	3BrZR	-7.75	0.56	*R*
20	3BrZS	-7.19		
21	4BrER	-7.04	3.13	*S*
22	4BrES	-10.17		
23	4BrZR	-6.92	0.42	*R*
24	4BrZS	-6.50		
25	2ClER	-7.17	4.03	*S*
26	2ClES	-11.20		
27	2ClZR	-7.12	3.98	*R*
28	2ClZS	-3.14		
29	3ClER	-6.20	5.29	*S*
30	3ClES	-11.49		
31	3ClZR	-5.10	1.79	*S*
32	3ClZS	-6.89		
33	4ClER	-6.27	3.76	*S*
34	4ClES	-10.03		
35	4ClZR	-7.44	0.04	-
36	4ClZS	-7.40		

Docking results with OxdRE-M29A-L145A-A147G

Docking of PPOX derivatives for the triple point mutant of OxdRE was performed according to GDP2. The partial energy contribution was not determined. Red number indicates low docking quality.

Table 8: Docking result with OxdRE-3-M and all mono substituted chloro-, bromo- and fluoro-PPOX derivatives (as for the ΔΔG-values, the absolute values are given (without sign); for each specific *E*- or *Z*-aldoxime, these values were calculated from the ΔG-values of the two related enantiomers of this aldoxime).

#	*PPOX*	*ΔG/ kcal · mol⁻¹*	*ΔΔG/ kcal · mol⁻¹*	*Enantiomer*
1	2FER	-6.55	1.77	*S*
2	2FES	-8.32		
3	2FZR	-3.13	0.20	*R*
4	2FZS	-2.93		
5	3FER	-6.82	1.87	*S*
6	3FES	-8.69		
7	3FZR	-2.53	1.00	*S*
8	3FZS	-3.53		
9	4FER	-7.12	1.54	*S*
10	4FES	-8.66		
11	4FZR	-2.43	1.40	*S*
12	4FZS	-3.83		
13	2BrER	-2.76	2.34	*S*
14	2BrES	-5.10		
15	2BrZR	-9.55	6.77	*R*
16	2BrZS	-2.78		
17	3BrER	-5.40	1.84	*S*
18	3BrES	-7.24		
19	3BrZR	-8.16	1.66	*R*
20	3BrZS	-6.50		
21	4BrER	-4.99	0.24	*S*
22	4BrES	-5.23		
23	4BrZR	-7.37	4.79	*R*
24	4BrZS	-2.58		
25	2ClER	-3.82	1.09	*S*
26	2ClES	-4.91		
27	2ClZR	-9.74	9.89	*R*
28	2ClZS	0.15		
29	3ClER	-3.64	6.62	*S*
30	3ClES	-10.26		
31	3ClZR	-6.60	1.47	*R*
32	3ClZS	-5.13		
33	4ClER	-2.16	6.24	*S*
34	4ClES	-8.40		
35	4ClZR	-4.12	1.69	*R*
36	4ClZS	-2.43		

Correlation of Enantioselectivity and ΔΔG-values

The energy values, which we determined via molecular modeling were used to calculate the E-value. The resulting E-values from the biotransformation were also transformed into the corresponding ΔΔG-values. Table 9 shows the calculated E-values from the energy values (ΔΔG) determined during docking experiments. Table 10 shows the energy values (ΔΔG) that were determined on the basis of the E values of the biotransformations

Table 9: Calculation of E-values from the determined energy values resulting from the *in silico* experiments.

	WT		*L145F*		*3M*	
	ΔΔG/ kcal · mol^{-1}	E-value	ΔΔG/ kcal · mol^{-1}	E-value	ΔΔG/ kcal · mol^{-1}	E-value
2FE	1870	29	3640	696	1730	22
2FZ	1630	19	2320	65	190	1
3FE	1980	35	2900	184	1870	29
3FZ	1510	15	70	1	1000	6
4FE	2650	117	3850	1016	1540	16
4FZ	930	5	1350	11	1340	11

Table 10: Calculation of energy values from the determined E-values resulting from the biotransformations.

	WT		*L145F*		*3M*	
	E-value	ΔΔG/ kcal · mol^{-1}	E-value	ΔΔG/ kcal · mol^{-1}	E-value	ΔΔG/ kcal · mol^{-1}
2FE	112	2623	302	3175	75	2401
2FZ	9	1222	20	1666	4	771
3FE	146	2771	309	3188	16	1542
3FZ	9	1222	8	1156	2	385
4FE	44	2104	64	2312	22	1719
4FZ	4	771	11	1333	1	0

6 Biotransformations

6.1 Activity assay

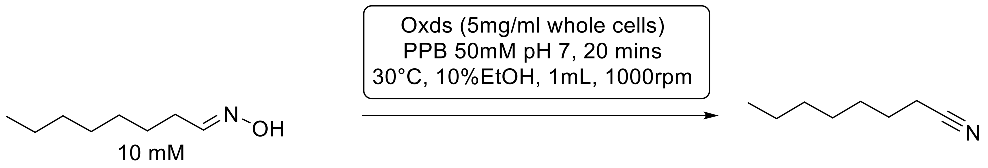

The activity assay was performed with whole cells of Oxd -variants in 5 mg/mL solution. First, the buffer (800 µL, PPB 50 mM, pH 7) was placed into a 2 mL Eppendorf vial. Then the substrate solution of octanal oxime in ethanol (100 µL, 100 mM) was added to the buffer to an end concentration of 10 mM octanal oxime and 10% ethanol. The temperature was arranged to 30°C. Afterwards the enzyme as whole cell solution was added to the reaction mixture. The reaction solution was stirred for 20 min at 1000 rpm. The reaction was quenched and extracted with 800 µL of ethyl acetate. The organic phase was analyzed via GC.

Entry	*Enzyme*	*Conversion / %*	*Specific activity/ mU/mg*
1	OxdRE-WT	68	68
2	OxdRE-L145F	52	52
3	OxdRE-10-(3M)<br>M29A/L145A/A147G	5	5
4	OxdRE-10-(3G)<br>M29G/L145G/A147G	0	0

6.2 Biocatalytic chiral nitrile synthesis

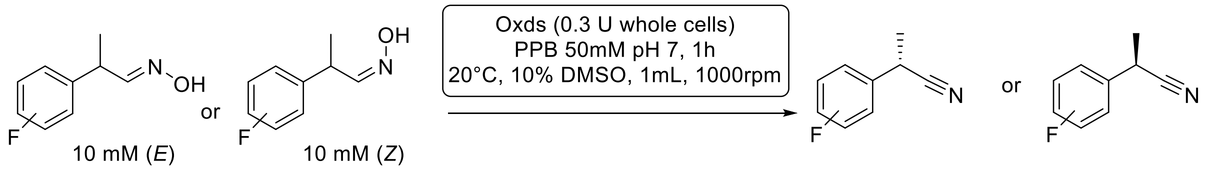

The biotransformation was performed with whole cells of Oxd -variants. First the buffer (PPB 50 mM, pH 7) was placed into a 2 mL Eppendorf vessel. Then the substrate solution, containing a FPPOX derivate in DMSO (100 µL, 100 mM), was added to the buffer to an end concentration of 10 mM FPPOX and 10% DMSO. The temperature was arranged to 20°C. Afterwards the enzyme as whole cell solution was added to the reaction mixture (0.3 U). The reaction solution was stirred for 1h min at 1000 rpm. The reaction was quenched and extracted with 800 µL of cyclohexane. The organic phase was analyzed via HPLC.

Table 11: Biotransformation of OxdRE-WT, OxdRE-L145F and OxdRE-3M with FPPOX derivatives as isolated isomers. Double point measurement and mean values. The E-value was calculated with the mean values.

OxdRE	*PPOX*	*Conv.1 /%*	*Conv.2 /%*	*Conv. Mean /%*	*Ee1 / %*	*Ee2 / %*	*Ee mw/ %*	*E-value*
WT	2FE (99/1)	43	47	45	96	96	96	112
WT	2FZ (1/99)	52	47	50	65	65	65	9
WT	3FE (99/1)	48	49	48	96	97	96	146
WT	3FZ (10/90)	52	51	52	64	65	64	9
WT	4FE (98/2)	35	34	34	93	93	93	44
WT	4FZ (3/97)	38	26	32	55	54	54	4
L145F	2FE (99/1)	28	31	29	>99	>99	>99	302
L145F	2FZ (1/99)	46	45	46	83	83	83	20
L145F	3FE (99/1)	33	29	31	>99	>99	>99	309
L145F	3FZ (10/90)	28	34	31	69	70	70	8
L145F	4FE (98/2)	19	25	22	97	95	96	64
L145F	4FZ (3/97)	18	26	22	81	80	80	11
3M	2FE (99/1)	45	46	45	94	95	94	75
3M	2FZ (1/99)	53	54	54	39	42	40	4
3M	3FE (99/1)	25	24	25	83	87	85	16
3M	3FZ (10/90)	45	50	48	66	32	17	2
3M	4FE (98/2)	40	39	39	84	86	85	22
3M	4FZ (3/97)	50	51	50	11	08	10	1

7 Analytical data

7.1 NMR

NMR spectra were recorded on a Bruker Avance III 500 at a frequence of 500 MHz (^1H). The chemical shift *δ* is given in ppm and referenced to the corresponding solvent signal ($CDCl_3$ or $(CD_3)_2SO$). The spectra were processed with MestReNova.

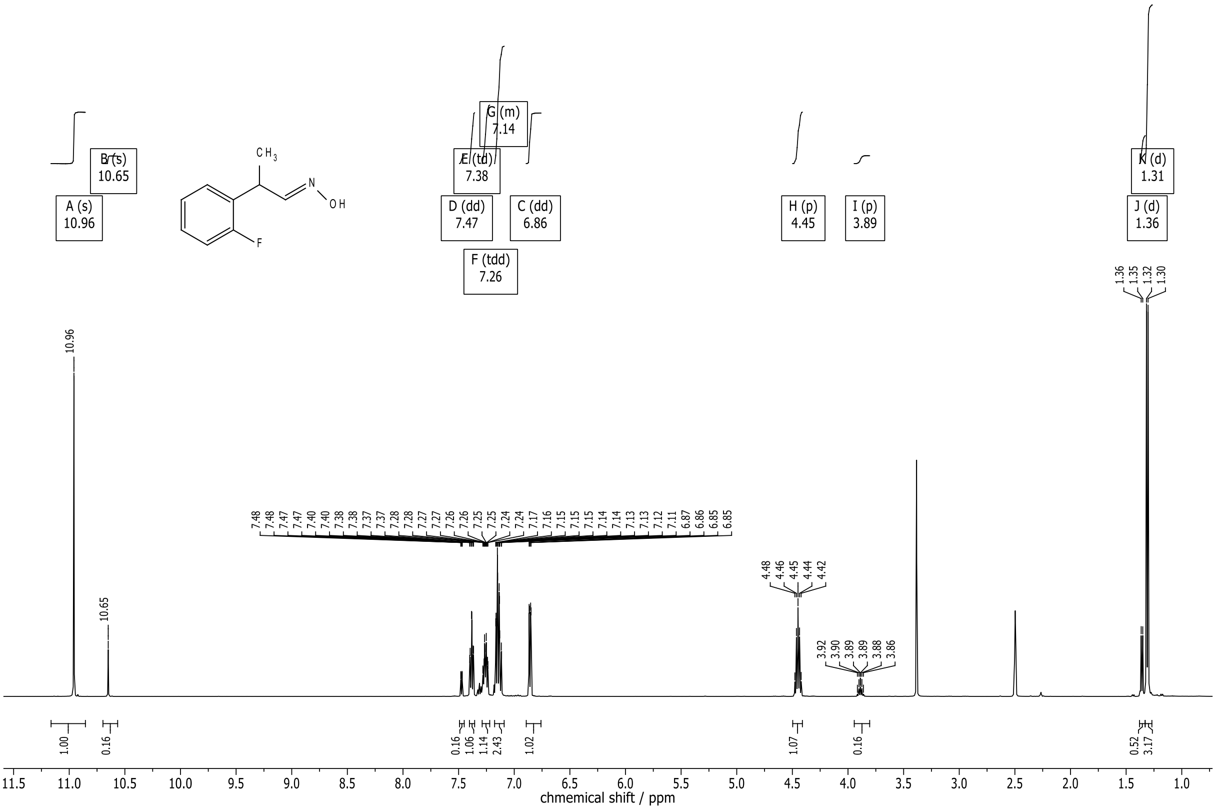

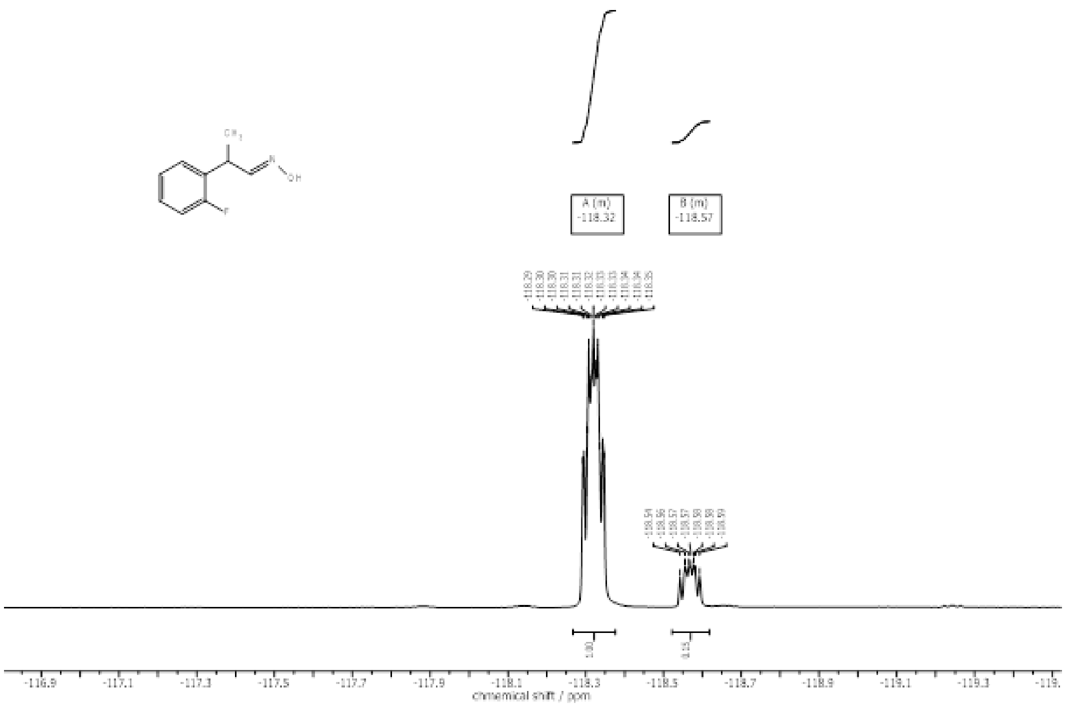
A (m)
-118.32
B (m)
-118.57
1.00
0.15
chmemical shift / ppm

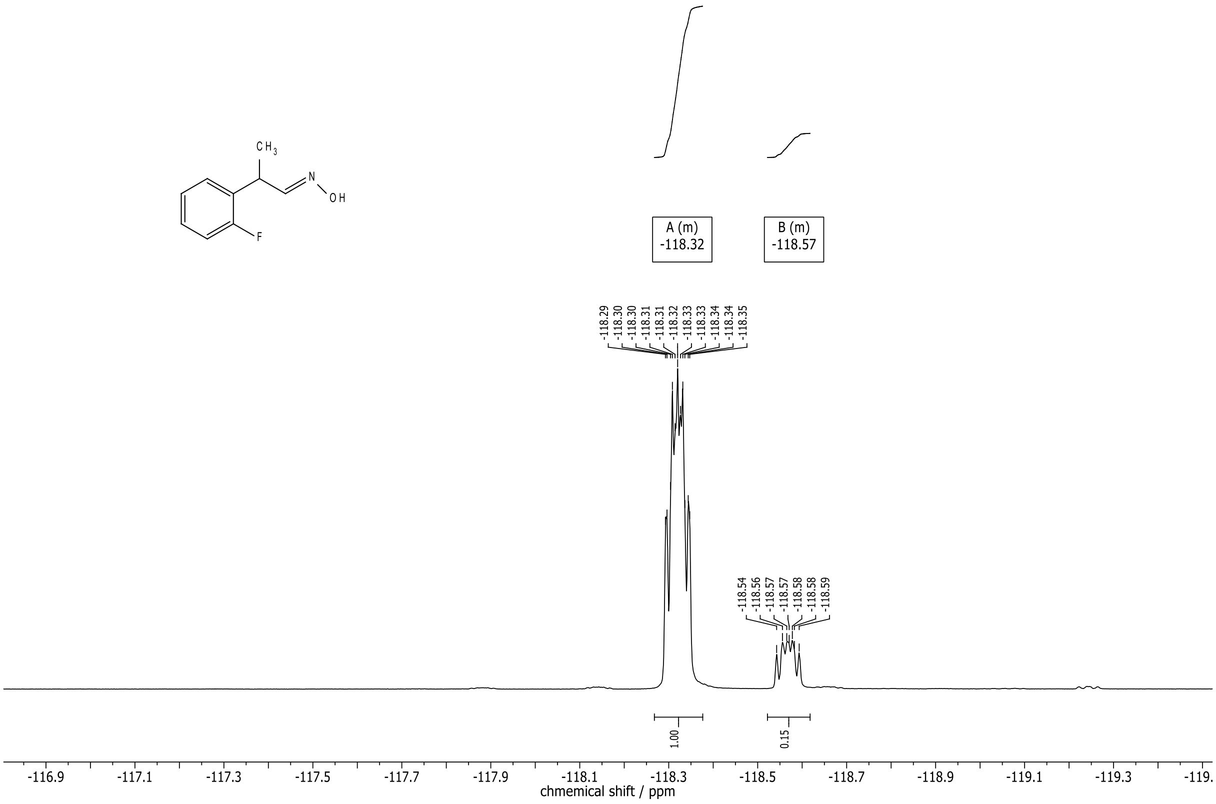
CH3
N
OH
F
A (m)
-118.32
B (m)
-118.57
-118.29
-118.30
-118.30
-118.31
-118.31
-118.32
-118.33
-118.33
-118.34
-118.34
-118.35
-118.54
-118.56
-118.57
-118.57
-118.58
-118.58
-118.59
1.00
0.15
-116.9
-117.1
-117.3
-117.5
-117.7
-117.9
-118.1
-118.3
-118.5
-118.7
-118.9
-119.1
-119.3
-119.
chmemical shift / ppm

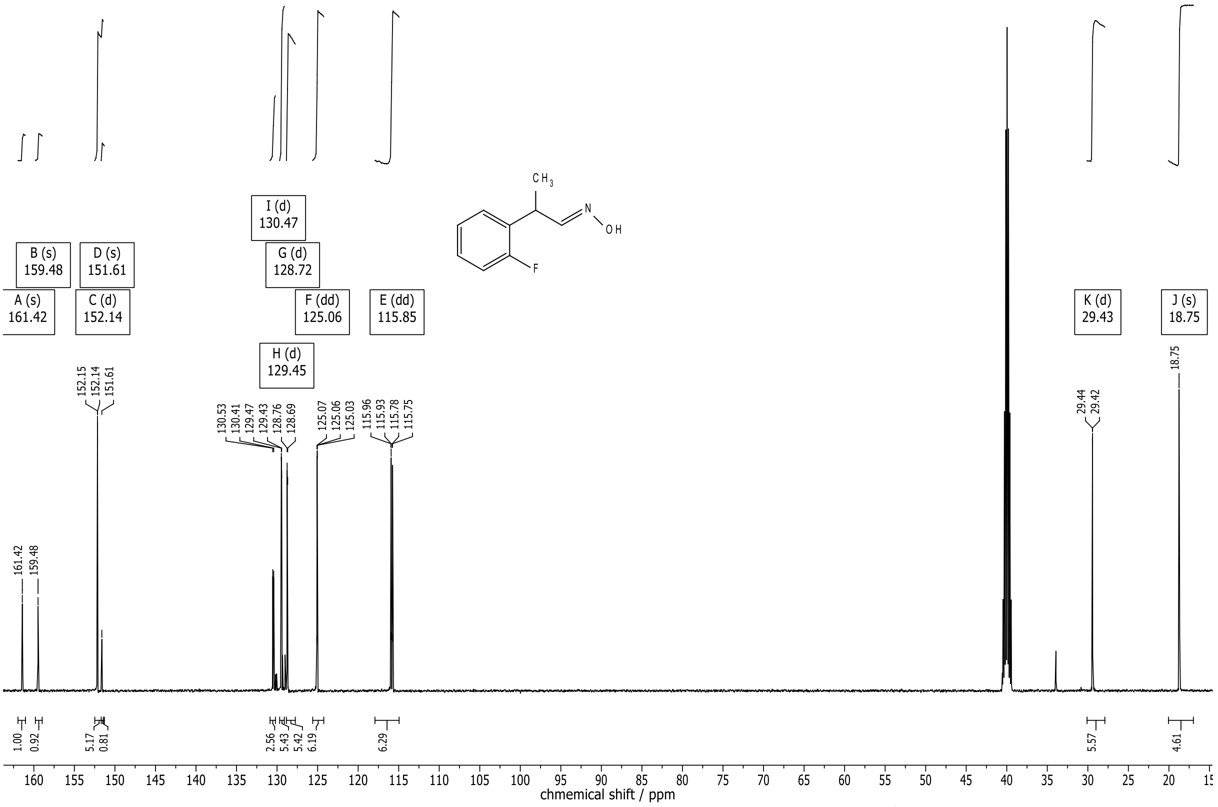
A (s)
161.42
B (s)
159.48
C (d)
152.14
D (s)
151.61
E (dd)
115.85
F (dd)
125.06
G (d)
128.72
H (d)
129.45
I (d)
130.47
J (s)
18.75
K (d)
29.43
chmemical shift / ppm

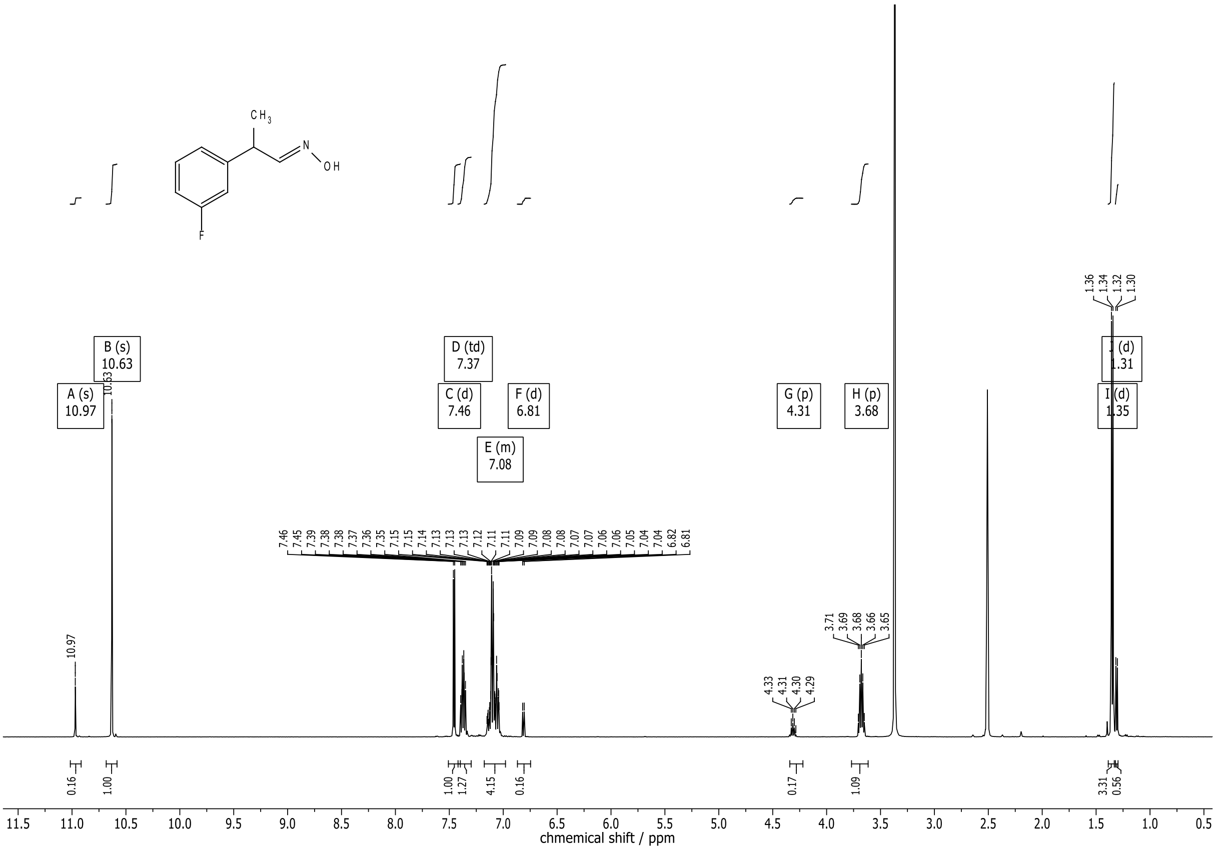
A (s)
10.97
B (s)
10.63
C (d)
7.46
D (td)
7.37
E (m)
7.08
F (d)
6.81
G (p)
4.31
H (p)
3.68
chmemical shift / ppm

^{19}F NMR (471 MHz, DMSO-d_6) δ -113.13 (ddd, J = 10.6, 8.8, 6.3 Hz), -113.19 – -113.28 (m).

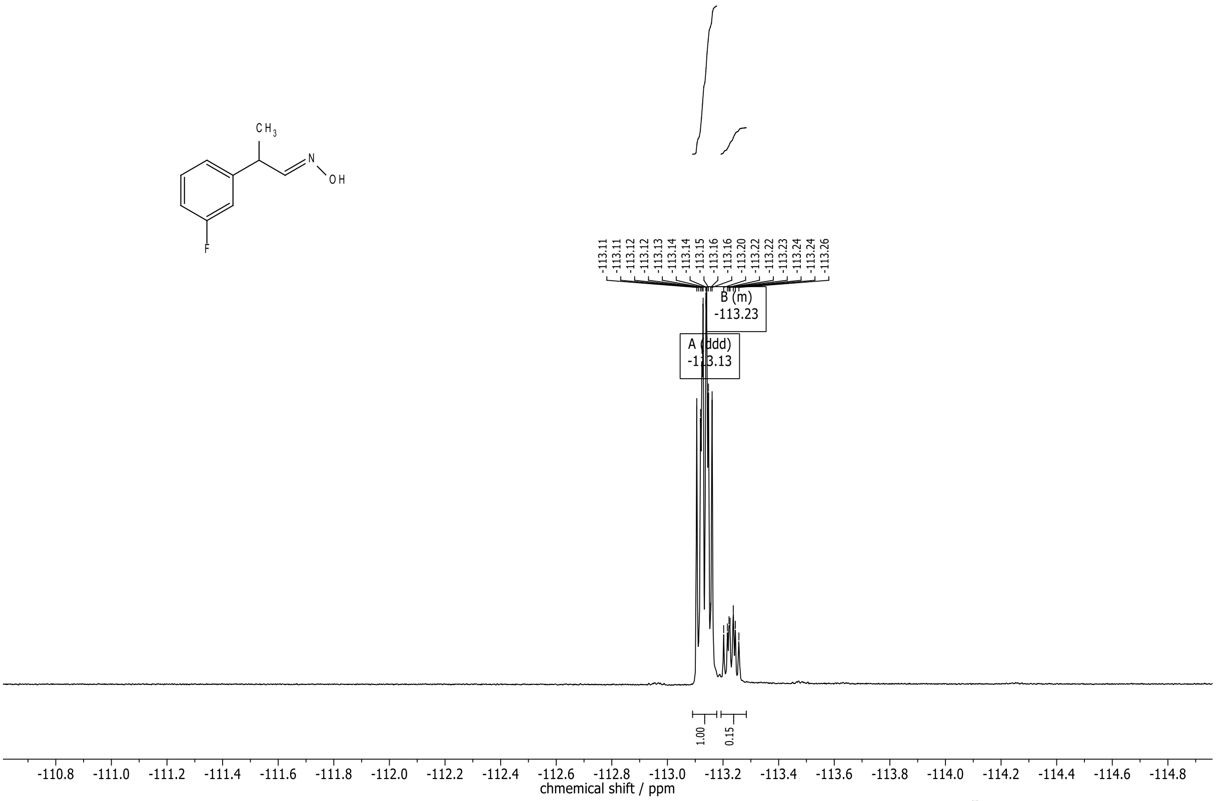

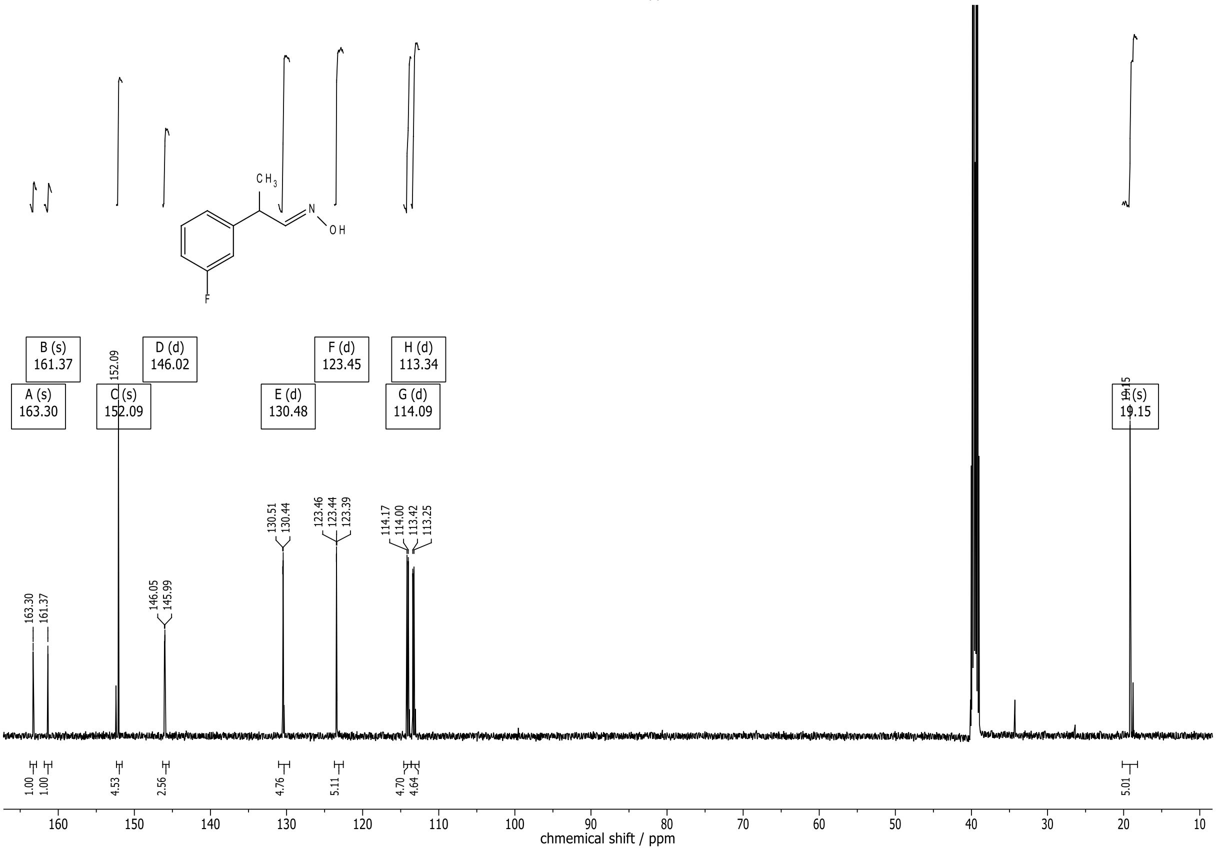

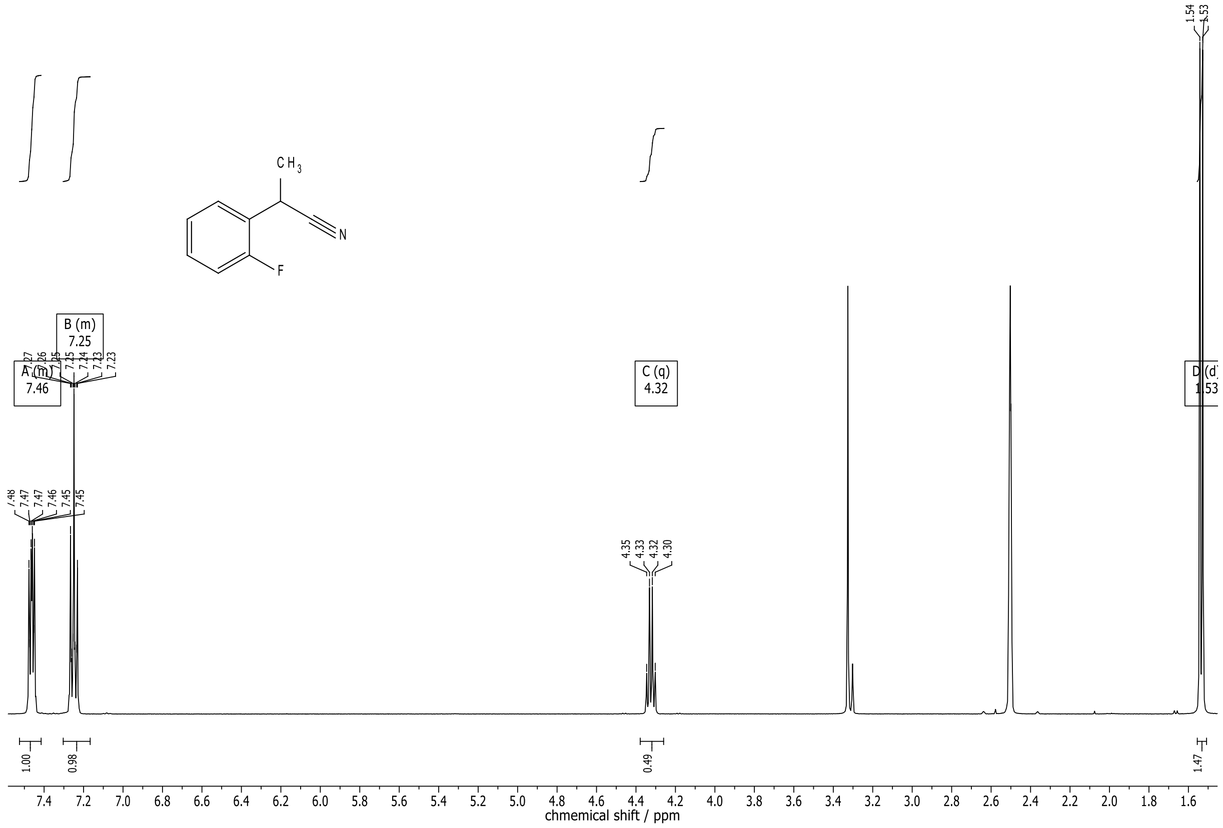
CH3
N
F
A (m)
7.46
B (m)
7.25
C (q)
4.32
D (d)
1.53
7.47
7.46
7.45
7.27
7.26
7.25
7.24
7.23
4.35
4.33
4.32
4.30
1.54
1.53
1.00
0.98
0.49
1.47
7.4
7.2
7.0
6.8
6.6
6.4
6.2
6.0
5.8
5.6
5.4
5.2
5.0
4.8
4.6
4.4
4.2
4.0
3.8
3.6
3.4
3.2
3.0
2.8
2.6
2.4
2.2
2.0
1.8
1.6
chmemical shift / ppm

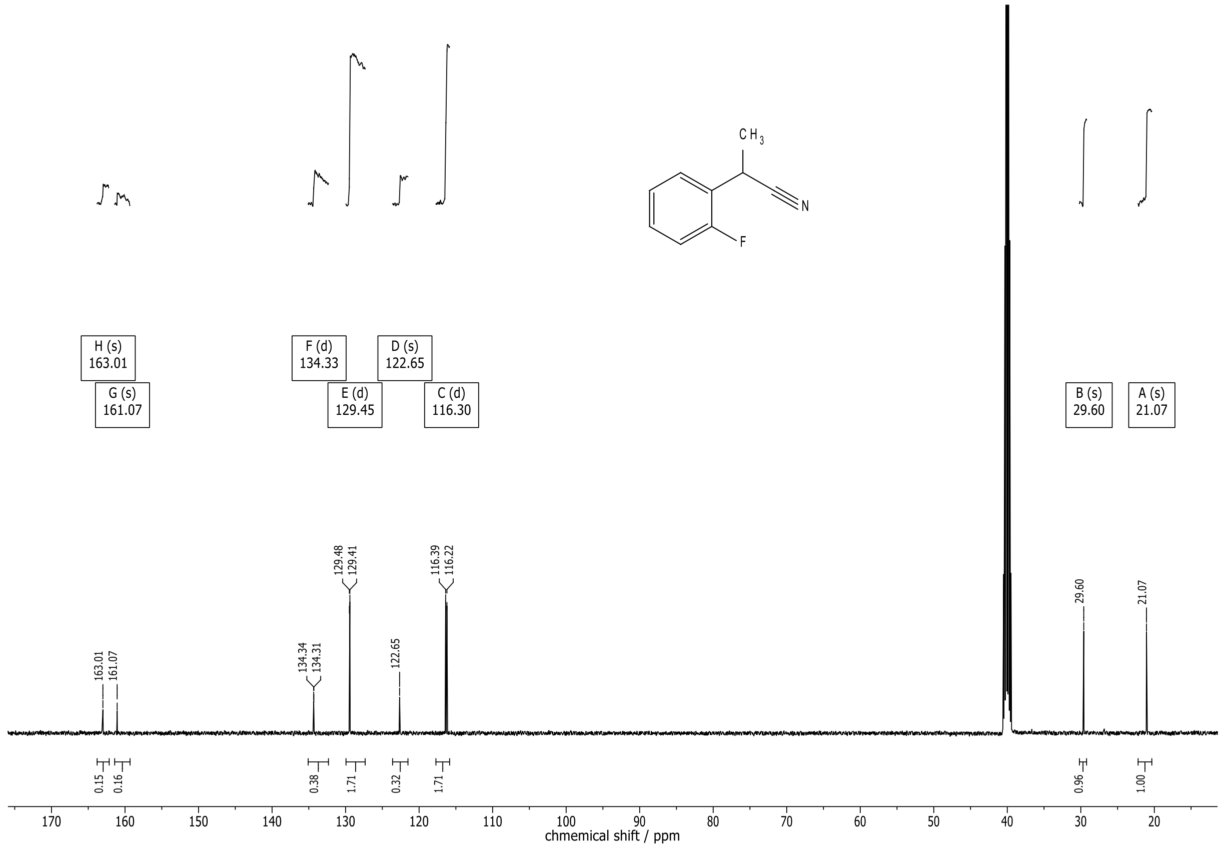
CH3
N
F
H (s)
163.01
G (s)
161.07
F (d)
134.33
E (d)
129.45
D (s)
122.65
C (d)
116.30
B (s)
29.60
A (s)
21.07
163.01
161.07
134.34
134.31
129.48
129.41
122.65
116.39
116.22
29.60
21.07
0.15
0.16
0.38
1.71
0.32
1.71
0.96
1.00
170
160
150
140
130
120
110
100
90
80
70
60
50
40
30
20
chmemical shift / ppm

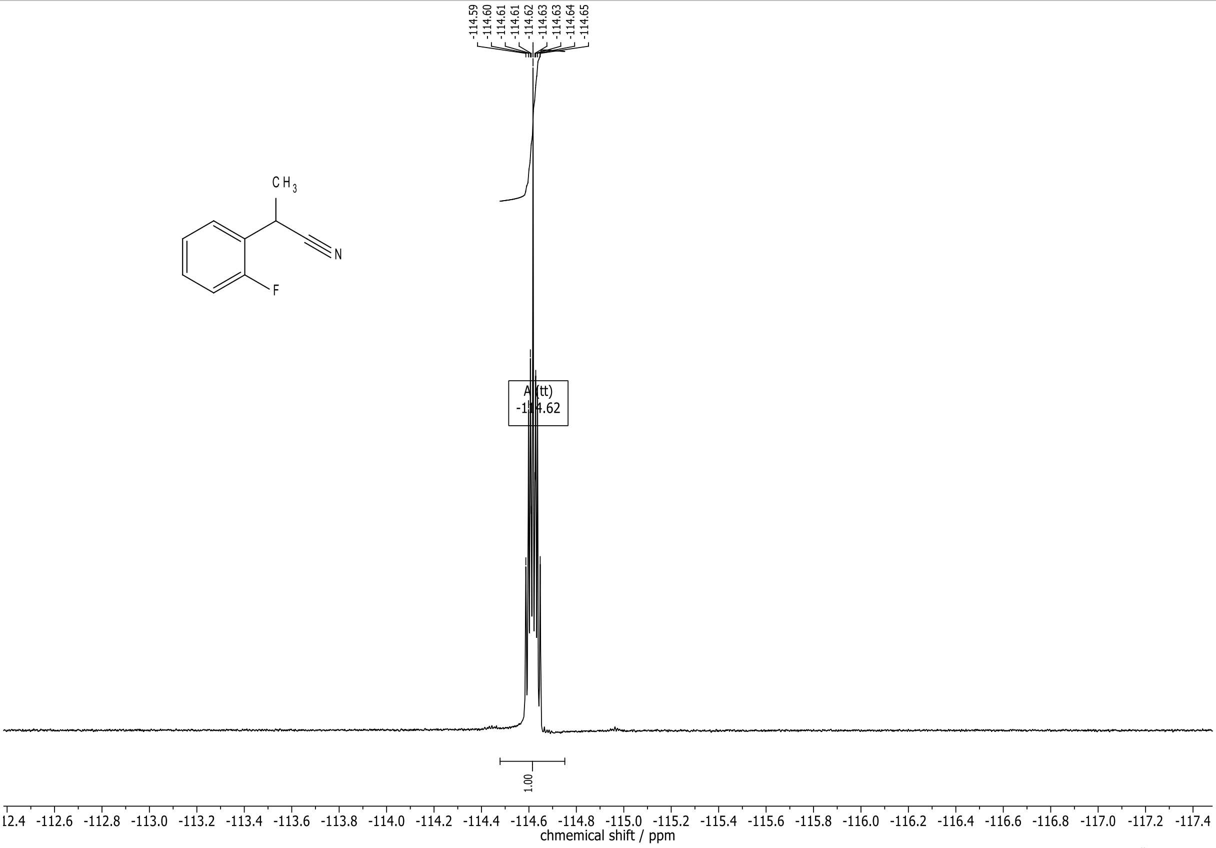
CH3
N
F
A (tt)
-114.62
1.00
chmemical shift / ppm

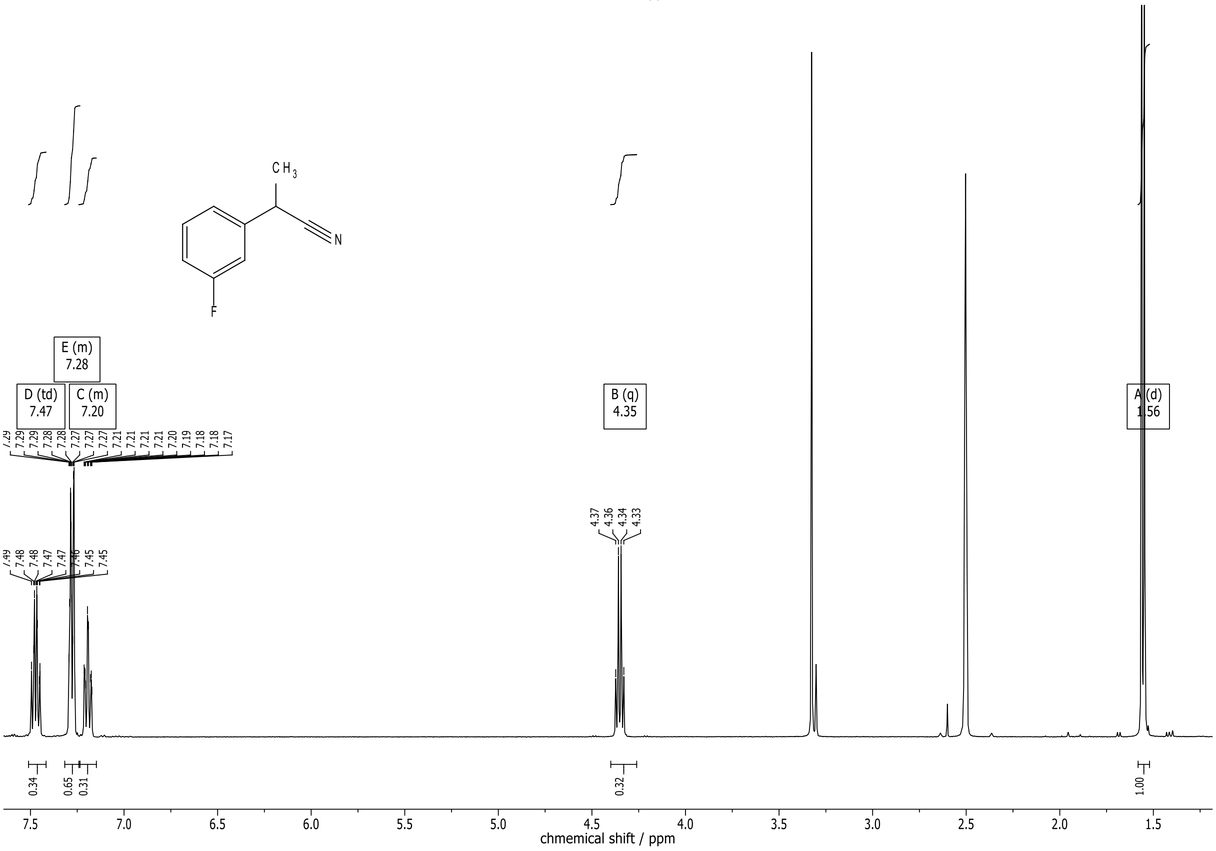
CH3
N
F
E (m)
7.28
D (td)
7.47
C (m)
7.20
B (q)
4.35
A (d)
1.56
0.34
0.65
0.31
0.32
1.00
chmemical shift / ppm

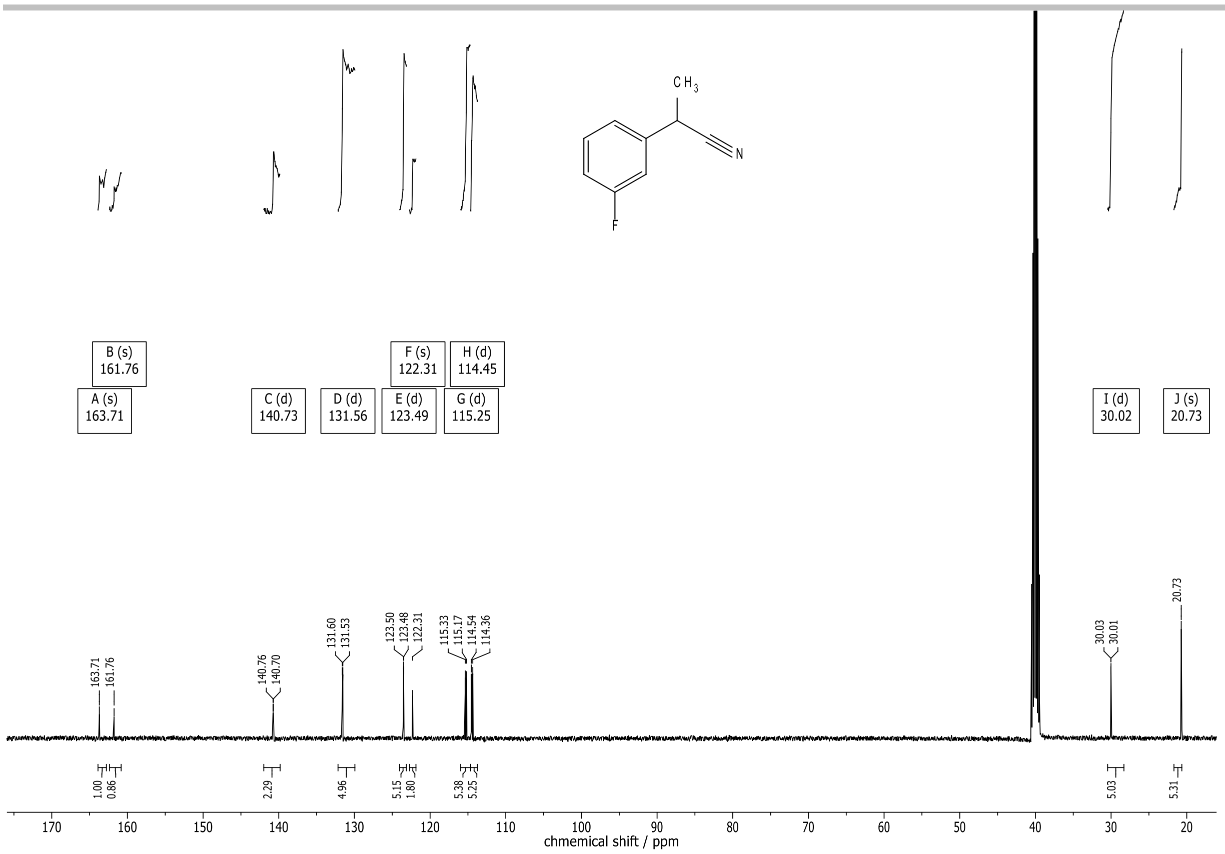
CH3
N
F
B (s) 161.76
A (s) 163.71
C (d) 140.73
D (d) 131.56
F (s) 122.31
E (d) 123.49
H (d) 114.45
G (d) 115.25
I (d) 30.02
J (s) 20.73
163.71
161.76
140.76
140.70
131.60
131.53
123.50
123.48
122.31
115.33
115.17
114.54
114.36
30.03
30.01
20.73
1.00
0.86
2.29
4.96
5.15
1.80
5.38
5.25
5.03
5.31
170 160 150 140 130 120 110 100 90 80 70 60 50 40 30 20
chmemical shift / ppm

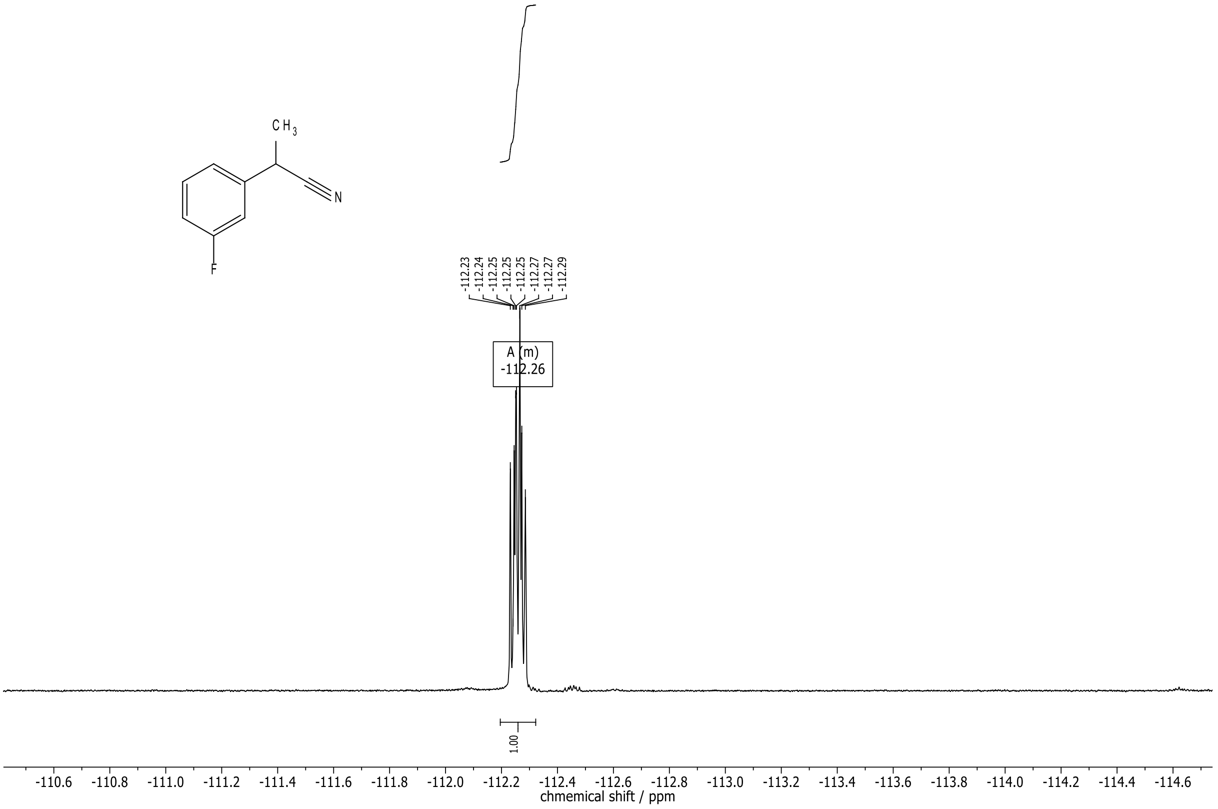
CH3
N
F
-112.23
-112.24
-112.25
-112.25
-112.25
-112.27
-112.27
-112.29
A (m) -112.26
1.00
-110.6 -110.8 -111.0 -111.2 -111.4 -111.6 -111.8 -112.0 -112.2 -112.4 -112.6 -112.8 -113.0 -113.2 -113.4 -113.6 -113.8 -114.0 -114.2 -114.4 -114.6
chmemical shift / ppm

EZ-separation:

The FPPOX derivatives were separated into the *E* and *Z*-isomers via automated column chromatography (Figure 2).

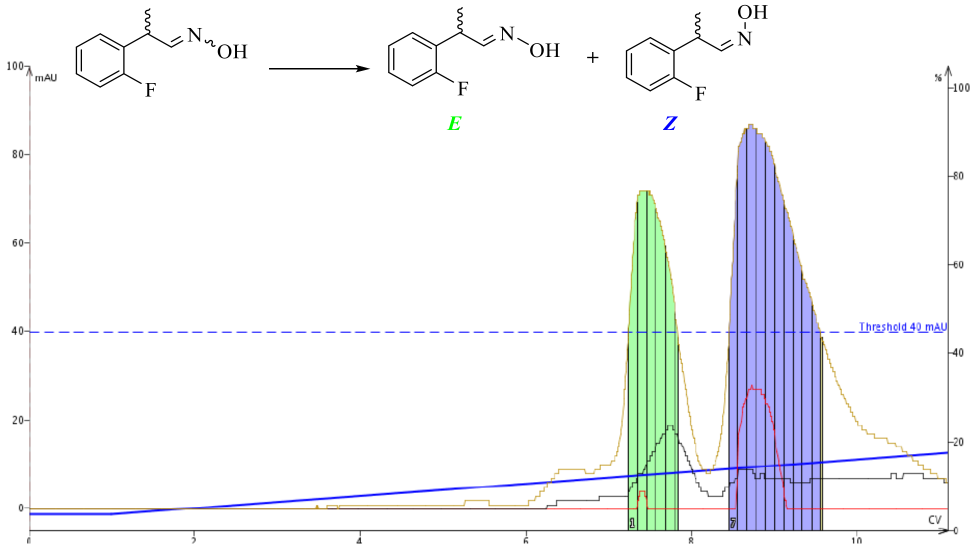

Figure 4:Chromatogram of the automated column chromatography for the *E/Z*-separation of 2-FPPOX as example.

NMR

Isomer purity was confirmed with ^{1}H-NMR in deuterated DMSO-d_6. The isomers are not stable in chloroform ($CDCl_3$).

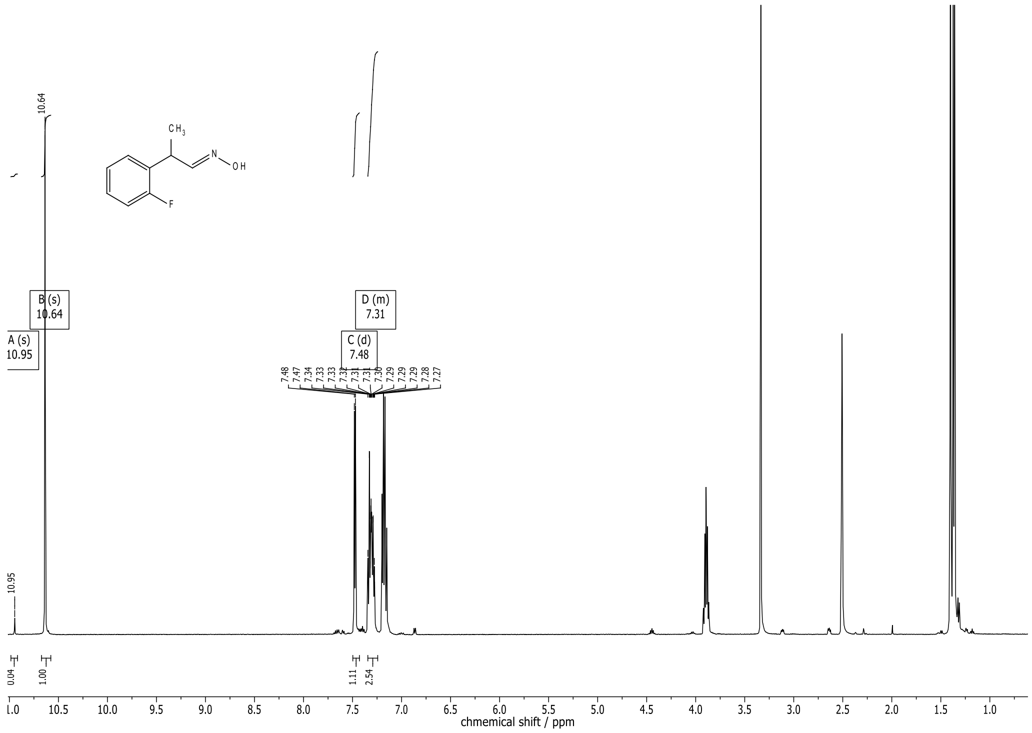

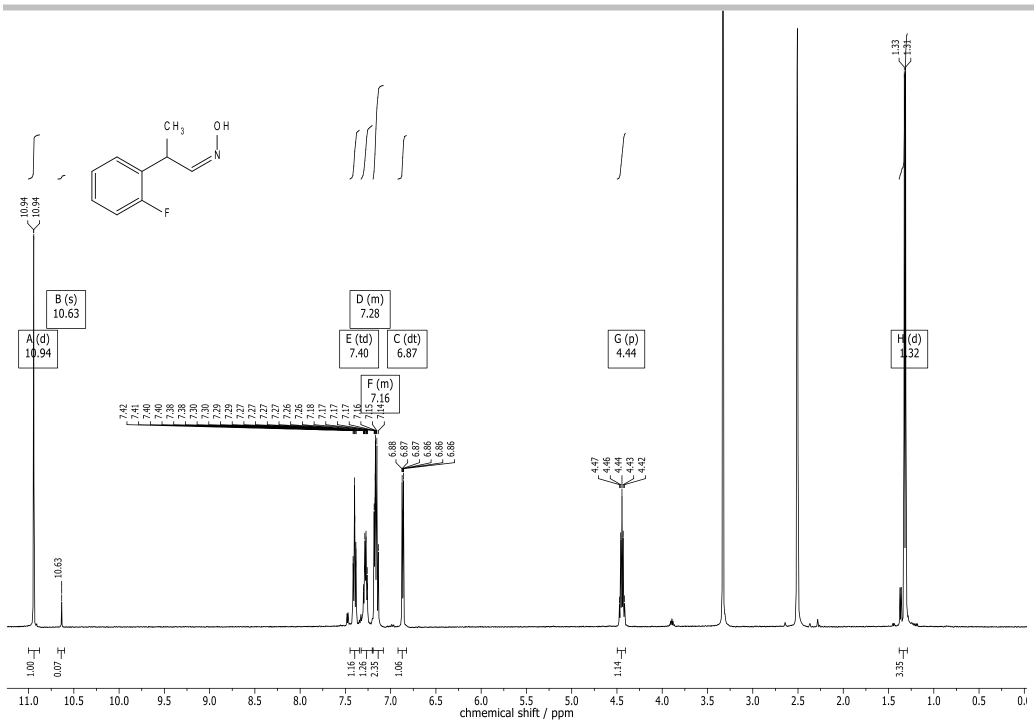
CH3
OH
N
F
A (d)
10.94
B (s)
10.63
C (dt)
6.87
D (m)
7.28
E (td)
7.40
F (m)
7.16
G (p)
4.44
H (d)
1.32
1.00
0.07
1.16
1.26
2.35
1.06
1.14
3.35
chmemical shift / ppm

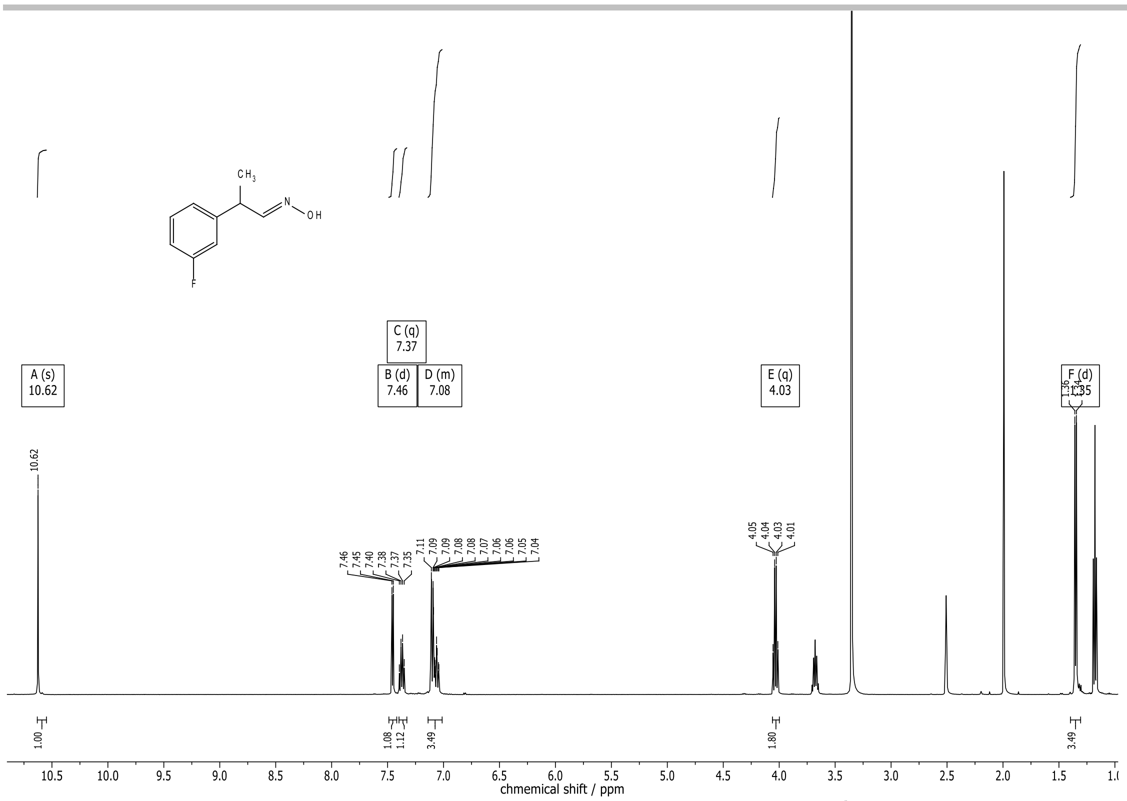
CH3
N
OH
F
A (s)
10.62
C (q)
7.37
B (d)
7.46
D (m)
7.08
E (q)
4.03
F (d)
1.35
chmemical shift / ppm

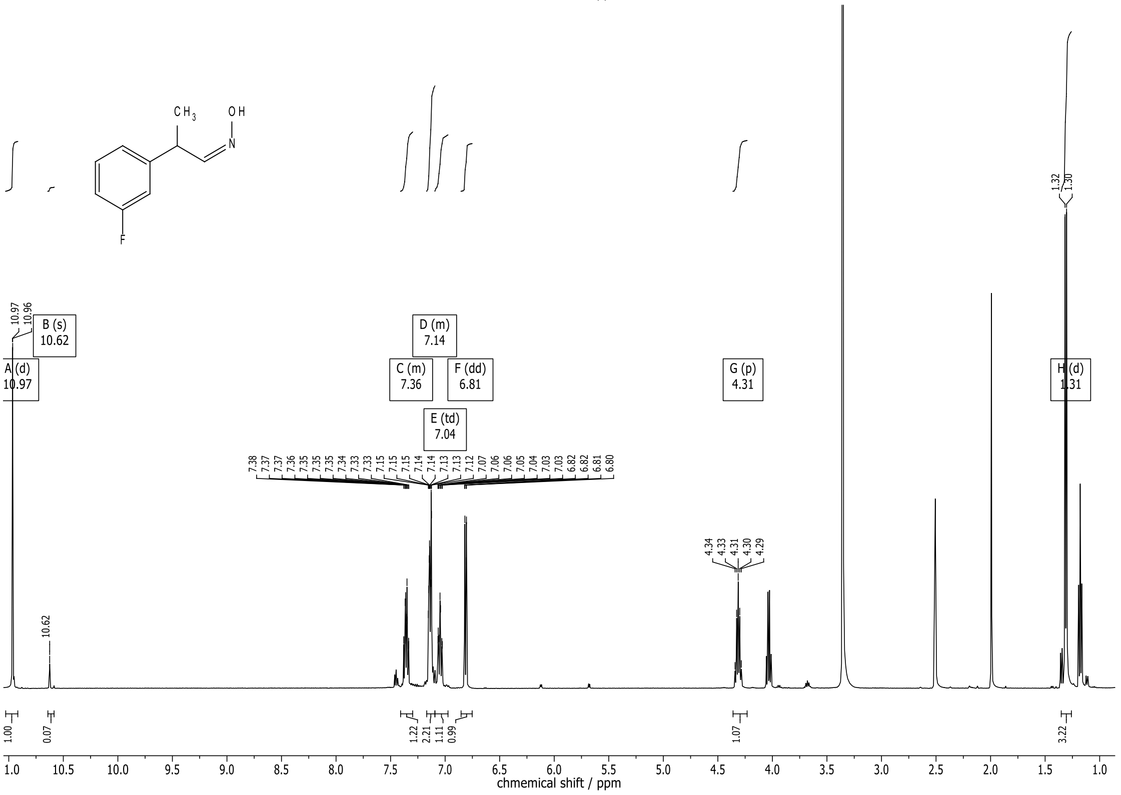
CH3
OH
N
F
B (s)
10.62
A (d)
10.97
D (m)
7.14
C (m)
7.36
F (dd)
6.81
E (td)
7.04
G (p)
4.31
H (d)
1.31
chmemical shift / ppm

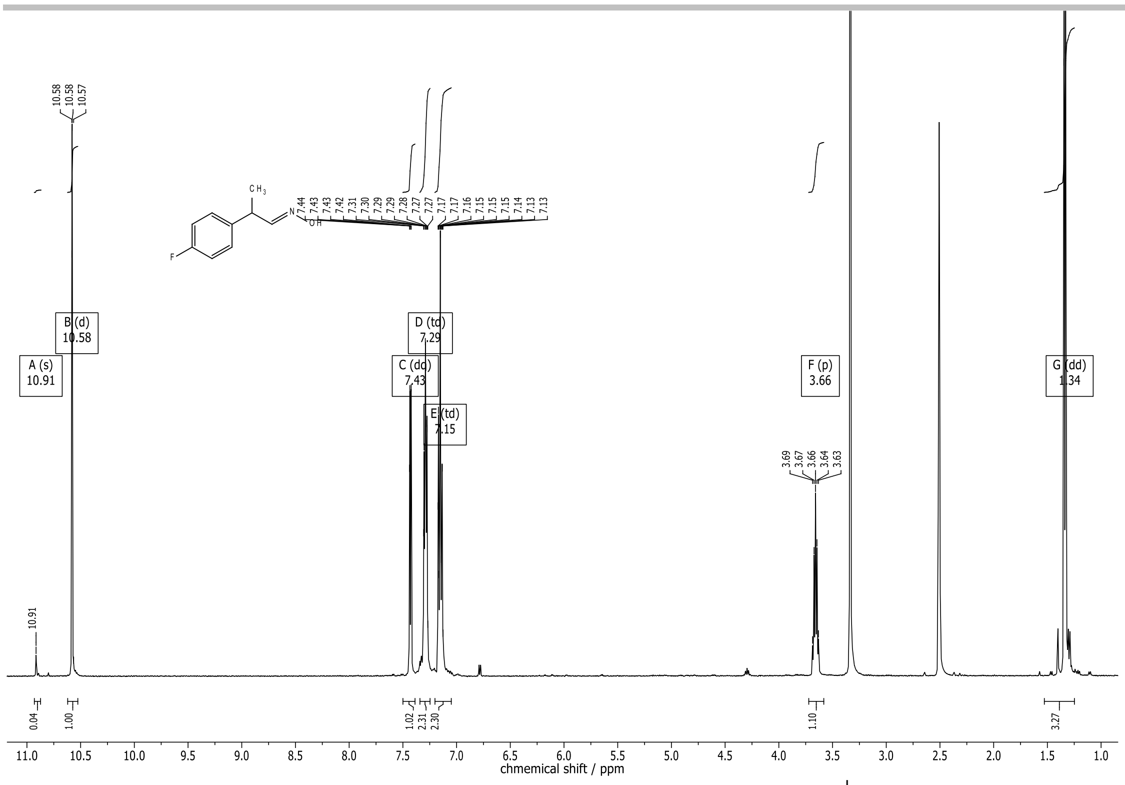
A (s)
10.91
B (d)
10.58
C (dd)
7.43
D (td)
7.29
E (td)
7.15
F (p)
3.66
G (dd)
1.34
chmemical shift / ppm

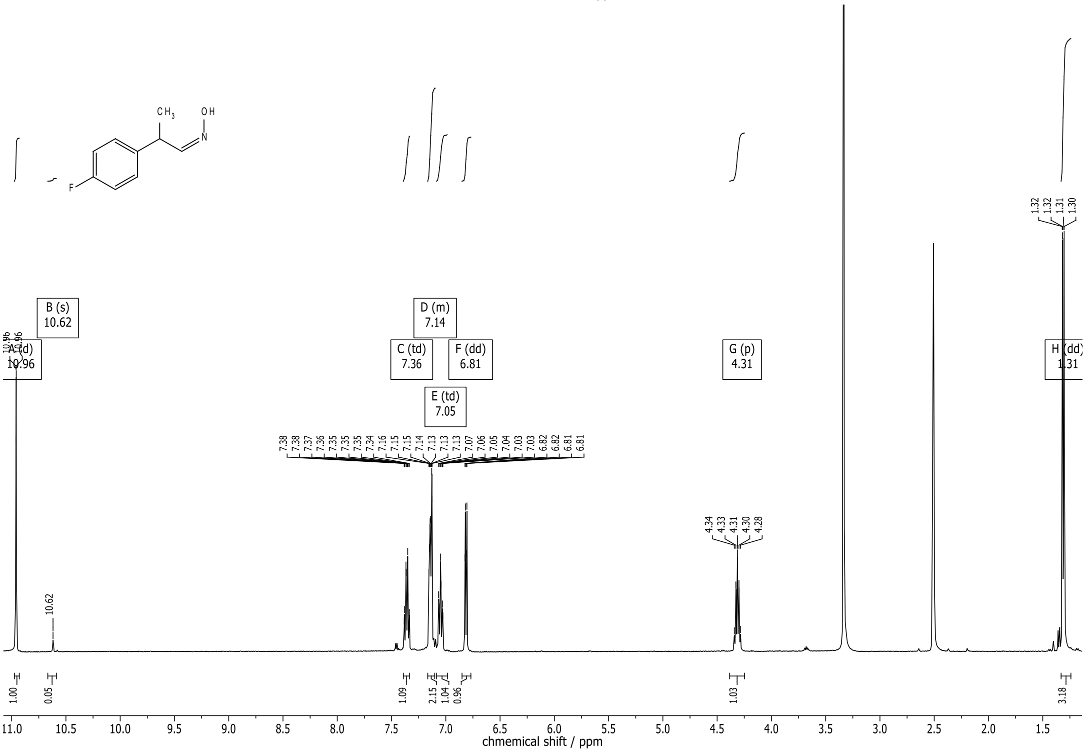
A (d)
10.96
B (s)
10.62
C (td)
7.36
D (m)
7.14
E (td)
7.05
F (dd)
6.81
G (p)
4.31
H (dd)
1.31
chmemical shift / ppm

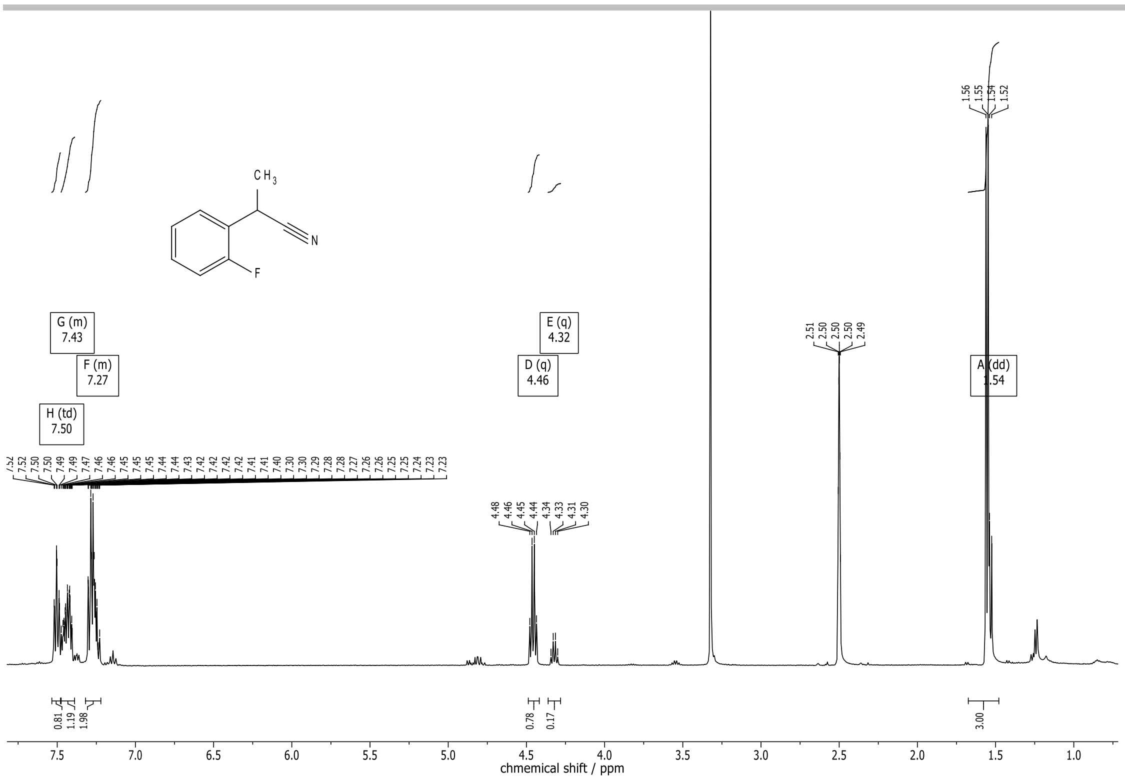

SFC-HPLC

The conversion was determined using a calibration curve for the SFC HPLC and using ^{1}H-NMR. The enantiomeric excess was determined by means of chiral, supercritical fluid chromatography (SFC-HPLC). The OB-H column from *Chiracel^{TM}* served as stationary phase. The mobile phase consisted of CO_2/isopropanol mixture (98:2, v/v). The method runs for 30 mins at 1.0 ml/min, the injection volume is 20 µL and the measurement runs at 20 ° C. The absorbance was measured at 210 nm.

MS and HRMS

The routine MS-spectra were measured via Esquire 3000. The Agilent Techn. 6220 TOF LCMS with ESI as ionization method was used for the accurate mass measurement.

References

[1] T. Betke, P. Rommelmann, K. Oike, Y. Asano, H. Gröger, *Angew. Chem. Int. Ed.* **2017**, *56*, 12361-12366.
[2] Molecular Operating Environment (MOE) 2019.01 (Chemical Computing Group ULC, Montreal, QC, Canada, 2019).
[3] J. Nomura, H. Hashimoto, T. Ohta, Y. Hashimoto, K. Wada, Y. Naruta, K.-I. Oinuma, M. Kobayashi, *Proc. Nat. Acad. Sci. USA* **2013**, *110*, 2810-2815.
